# Understanding the Role of *PIN* Auxin Carrier Genes under Biotic and Abiotic Stresses in *Olea europaea* L.

**DOI:** 10.3390/biology11071040

**Published:** 2022-07-11

**Authors:** Hélia Cardoso, Catarina Campos, Dariusz Grzebelus, Conceição Egas, Augusto Peixe

**Affiliations:** 1 MED—Mediterranean Institute for Agriculture, Environment and Development & CHANGE—Global Change and Sustainability Institute, Instituto de Investigação e Formação Avançada, Universidade de Évora, Pólo da Mitra, Ap. 94, 7006-554 Évora, Portugal; mccampos@uevora.pt; 2 Department of Plant Biology and Biotechnology, Faculty of Biotechnology and Horticulture, University of Agriculture in Krakow, Al. 29 Listopada 54, 31-425 Krakow, Poland; d.grzebelus@urk.edu.pl; 3 Biocant-Biocant Park, Núcleo 04 Lote 8, 3060-197 Cantanhede, Portugal; cegas@biocant.pt; 4 Center for Neuroscience and Cell Biology, Rua Larga—Faculdade de Medicina, 1ºandar—POLO I, University of Coimbra, 3004-504 Coimbra, Portugal; 5 MED—Mediterranean Institute for Agriculture, Environment and Development & CHANGE—Global Change and Sustainability Institute, Departamento de Fitotecnia, Escola de Ciência e Tecnologia, Universidade de Évora, Pólo da Mitra, Ap. 94, 7006-554 Évora, Portugal; apeixe@uevora.pt

**Keywords:** auxin, PIN transporters, stress response, expression pattern, olive

## Abstract

**Simple Summary:**

The plant hormone auxin is involved in the majority of the processes related to plant development and growth and response to environmental constraints. The PIN proteins are a group of auxin transporters that are also involved in plant responses to stresses. This study describes the *PIN* gene family in the domesticated olive tree (*Olea europaea* subsp. *europaea* var. *europaea*) and its wild relative (*O. europaea* subsp. *europaea* var. *sylvestris*). Twelve and 17 *PIN* genes were identified for vars. *sylvestris* and *europaea*, respectively, being distributed across 6 subfamilies. Differences in the patterns of gene diversification and subfamilies expansion were observed across subfamilies. Genes encoding canonical OePIN proteins comprise six exons, while genes encoding non-canonical OePINs are composed of five exons, with implications for protein specificities and functionality. Expression analysis of *OePINs* from RNA-seq data showed that members from the subfamilies 1, 2, and 3 responded to abiotic and biotic stress factors. Our study shows the diversification of *PINs* in an important species such as the olive tree and highlights the importance of *PIN* genes on stress responses, contributing to a holistic understanding of the role of auxins in plants.

**Abstract:**

The PIN-FORMED (PIN) proteins represent the most important polar auxin transporters in plants. Here, we characterized the *PIN* gene family in two olive genotypes, the *Olea europaea* subsp. *europaea* var. *sylvestris* and the var. *europaea* (cv. ‘Farga’). Twelve and 17 *PIN* genes were identified for vars. *sylvestris* and *europaea*, respectively, being distributed across 6 subfamilies. Genes encoding canonical OePINs consist of six exons, while genes encoding non-canonical OePINs are composed of five exons, with implications at protein specificities and functionality. A copia-LTR retrotransposon located in intron 4 of *OePIN2b* of var. *europaea* and the exaptation of partial sequences of that element as exons of the *OePIN2b* of var. *sylvestris* reveals such kind of event as a driving force in the olive *PIN* evolution. RNA-seq data showed that members from the subfamilies 1, 2, and 3 responded to abiotic and biotic stress factors. Co-expression of *OePINs* with genes involved in stress signaling and oxidative stress homeostasis were identified. This study highlights the importance of *PIN* genes on stress responses, contributing for a holistic understanding of the role of auxins in plants.

## 1. Introduction

Auxin is a universal phytohormone that participates in multiple aspects of plant growth and development, including phloem and wood formation [1,2], fruit and root development [3,4,5], leaf formation [6], shoot elongation and embryo pattern development [7,8], flower abscission [9] and phototropism and gravitropism [3,10]. Additionally, auxin has also been used as a modulator of biological processes involving cell reprogramming events, like primary culture systems based on cell dedifferentiation and further growth [11], adventitious rooting [12], and somatic embryogenesis [13,14]. These last two processes are described as morphogenic responses to abiotic stress stimulus [15,16]. In fact, the involvement of auxin in plant responses to biotic and abiotic environmental stress factors has also been demonstrated in several studies [17,18,19].

Indole-3-acetic acid (IAA) is the main form of auxin in plants, presenting a coordinative signal in all biological processes coordinated by auxin. The involvement of auxin in the different aspects of plant growth and development, as well in plant stress response, depends on its cellular level, which directly depends on auxin metabolism (biosynthesis, degradation, and conjugation), long-distance transport, and directional cell-to-cell translocation [8]. Auxin is usually synthesized at the level of shoot apex or primordia of developing leaves and further transported to the target tissues through bulk flow via vascular tissues or direct polar auxin transport (PAT), a highly regulated activity that determines the acquisition of cellular auxin homeostasis dependent on auxins influx and efflux carriers [20]. Several auxin transporters belonging to PAT were previously described, like the AUXIN1/LIKE-AUX1 (AUX/LAX), PIN-FORMED (PIN), PIN-LIKES (PILS), ATP-binding cassette family B (ABCB)-P-glycoprotein (PGP), nitrate transporter 1.1 (NRT1.1), and WALLS ARE THIN 1 (WAT1) [21,22]. Nonetheless, recent reports have highlighted the key role of PIN transporters in providing directionality to the intercellular auxin flow work as influx and efflux carriers to facilitate auxin distribution within the cells [23,24]. Several reports have shown that the silencing of different *PIN* genes, leading to an abnormal auxin accumulation, strongly affects plant development [25,26,27].

The first study regarding *PIN* gene family characterization came from Paponov and co-authors [28], who reported eight members in *Arabidopsis thaliana* (L.) Heynh., each representing a different subfamily (*AtPIN1*, *AtPIN2*, *AtPIN3*, *AtPIN4*, *AtPIN5*, *AtPIN6*, *AtPIN7*, and *AtPIN8*). Recently, several studies revealed the *PIN* gene family structure across several plant species, including agronomically important crops like rice, maize, potato, soybean, and cotton [29,30,31,32,33]. In one of those reports, a new subfamily, named *PIN9*, was described in *Fabaceae* [29]. High heterogeneity in terms of subfamilies composition and radiation has been highlighted, and both characteristics seem to be species-dependent, going from four genes in *Marchantia polymorpha* L. to 23 genes in *Glycine max* (L.) Merr. [23].

*PIN* gene structure has also been explored in reports showing high structure conservation across genes belonging to the two main *PIN* groups, usually comprising five to six exons [32,34,35]. The extra exon is associated with the presence of a long sequence encoding a central hydrophilic intracellular loop (HL) with highly-conserved HC1–HC4 regions, about 35 motifs identified between amino- and carboxyl-terminal transmembrane domains, which form an auxin-translocation pore at the plasma membrane, exhibiting a prominent role in the directional, cell-to-cell auxin transport [36]. Members carrying long HL located on the central loop domain are considered canonical PIN proteins and are usually classified as PIN1–PIN4 and PIN7. In contrast, members carrying short HL are referred as non-canonical proteins and belong to PIN5, PIN8, and PIN9 subfamilies. Proteins belonging to these two subfamilies are located in the intracellular compartments, with an important role in the regulation of auxin exchange between cytosol and endoplasmic reticulum, contributing to the intercellular auxin homeostasis [19]. PIN6 is a member that, despite belonging to the long HL group, carries a reduced HL sequence and shows the dual location. Depending on the phosphorylation state of this auxin carrier, it can be located at the plasma membrane or endoplasmic reticulum [37].

The analysis of *PINs* on perennial woody plant species has shown that *PIN1*-members are associated with dwarfing tree phenotype, plant growth vigor, root gravitropism, embryo development, fruit abscission, and maintenance of PAT in the cambial region [3,5,10,35,38,39,40]. *PIN2* was associated with the initiation of the functional meristem, *PIN3* with leaf morphogenesis, *PIN4* with fruit development [38], *PIN7* with primary growth by being more expressed at internodes [40], and *PIN9* with promoting lateral root formation [4]. From non-canonical *PINs*, a single report is known on *PIN8*, revealing its association with dwarfing [39].

Besides the involvement of the different *PIN* members in a range of biological processes associated with plant growth and development, some studies have also explored their involvement in plant stress responses. For instance, *PINs*’ expression in *Arabidopsis* was altered in response to the infection with nematodes [41]. Pasternak et al. [42] reported a decrease in *PIN1* and *PIN3* in *Arabidopsis* in response to oxidative stress caused by alloxan, and Shibasaki et al. [43] reported a down-regulation of *PIN2* and *PIN3* in response to cold. The involvement of *PINs* in de novo morphogenesis processes has also been highlighted. Hakman and co-authors [7] reported the involvement of *PaPIN1* in the development of somatic embryos in *Picea abies* (L.) H. Karst., in a way similar to that of zygotic embryos. Additionally, the involvement of *PIN* members, in particular *PIN1* and *PIN2*, in the process of adventitious root formation was also reported in *Eucalyptus globulus* Labill [44] and *Olea europaea* L. [45]. Nevertheless, no information regarding the characterization of olive *PINs* has been available.

In the present study, we performed a genome-wide analysis of the *PIN* gene family in *O. europaea* subsp. *europaea* var. *europaea* and var. *sylvestris*. To our knowledge, it is the first study that is focused on the identification of *OePIN* members and their characterization at the genomic and transcriptomic levels. Besides the phylogenetic and structural analysis of gene members, which contribute to the understanding of the molecular structure and evolution of the *PIN* family in plants, expression patterns of the different *PIN* members were also investigated. The availability of olive RNAseq data from different Bioprojects in response to a range of stresses (cold, wounding, and infection with *Verticillium dahlia* Kleb.) allowed reanalysis of the data focused on the *PIN* members. The results provide a solid foundation for future olive *PIN* gene-related studies considering plant response to low-temperature stress conditions, wounding, and infection with *V. dahliae*.

## 2. Materials and Methods

### 2.1. Identification of OePIN Genes and Phylogenetic Analysis

To determine the number of genes that compose the *PIN* family in *O. europaea* subsp. *europaea*, and retrieve all olive *AtPIN*-homologous sequences, a Blast search was performed using the olive genome database (http://denovo.cnag.cat/olive (accessed on 30 November 2017), Oe6 browser), available for the var. *europaea* (cv. ‘Farga’). *PIN* sequences retrieved from *A. thaliana* were used as queries and the resulting sequences as secondary queries. Subsequently, a Blastn analysis was performed at the NCBI (National Center for Biotechnology Information, https://www.ncbi.nlm.nih.gov/ (accessed on 7 December 2018)) to verify the homology with *PIN* sequences. Additionally, *PIN* sequences were also identified in the genome of *O. europaea* subsp. *europaea* var. *sylvestris*, the wild relative of cultivated olive tree (also known as *oleaster*), which has more complete information regarding gene location at the chromosome level (available at https://www.ncbi.nlm.nih.gov/genome/?term=Olea+europaea+var.+sylvestris+genome (accessed on 1 October 2018)).

A comparison was performed among the olive translated PIN sequences and 120 PIN sequences from 11 eudicot plant species to classify the identified sequences in *O. europaea*, for both varieties, *europaea*, and *sylvestris* (see details in Supplementary Materials Table S1). Sequences were retrieved from Phytozome (https://phytozome.jgi.doe.gov/pz/portal.html (accessed on 7 December 2018)) using *AtPIN* sequences as queries and the resulting sequences as secondary queries or by directly using published accession numbers of *PIN* gene family members in diverse plant species.

The retrieved sequences were aligned in MUSCLE (http://www.ebi.ac.uk/Tools/msa/muscle/ (accessed on 10 December 2018)) following the default settings to generate an output Pearson/FASTA file. A phylogenetic tree was constructed with MEGA 7 [46] using the Neighbor-Joining (NJ) method [47]. The inferred tree was tested by bootstrap analysis using 1000 replicates, “number of differences” as the substitution model and “pairwise deletion” for gaps/missing data treatment. A graphical view of the midpoint rooted tree was edited in the Fig Tree v14.0 software ([48], Edinburgh, UK) (http://tree.bio.ed.ac.uk/software/figtree/ (accessed on 10 December 2018)).

### 2.2. In Silico Analysis of PIN Structure and Identification of Regulatory Elements within Genic Regions

The information about gene exon-intron structure information for *OePIN* genes was assessed by Splign (https://www.ncbi.nlm.nih.gov/sutils/splign/splign.cgi?textpage=online&level=form (accessed on 6 March 2018)).

Sequences of all variants of these genes were self-aligned using Blast2Seq tool at NCBI (blast.ncbi.nlm.nih.gov (accessed on 28 August 2019)) to identify autonomous long terminal repeat retrotransposons (LTR-RTs) within the *PIN* genic regions. Sequences were also used as queries to search the GenBank nucleotide database restricted to the *Olea* genus (taxid:4145) and search for the most similar elements in the RepBase using Censor [49]. Target site duplications (TSDs) were identified manually. Additional manipulations and alignments of genomic variants and corresponding mRNAs were performed in BioEdit 7.2.5 [50]. The age of insertion of a copia-LTR retrotransposon in the *PIN2b* gene was estimated by comparing sequences of the two long terminal repeats (LTRs), essentially as proposed by Barghini and co-authors [51], using a substitution rate of 3.6 × 10^−9^.

### 2.3. Identification of PIN Conserved Protein Structural Features

Protein length, molecular weight, isoelectric point, and hydrophobic amino acids’ composition were determined with the EditSeq package from Lasergene 7 (DNASTAR, Madison, WI, USA). Transmembrane helices and the topology of proteins were predicted at the THMM automatic server (https://services.healthtech.dtu.dk/service.php?TMHMM-2.0 (accessed on 30 July 2019)). Protein subcellular localization and signal peptide identification were predicted using PSORT—Prediction of Protein Localization Sites, version 6.4 [52] (Tokyo, Japan) (http://psort1.hgc.jp/form.html (accessed on 30 July 2019)).

Conservation of the principal PIN protein structural features across all OePIN members identified was investigated by aligning the 17 previously identified OePIN sequences. The motifs or regulatory elements were identified based on previous reports using the CLC Main Workbench 7.5.1 software (ClCbio, Aarhus, Denmark) [23,53].

### 2.4. Mapping Procedures and Identification of Differentially Expressed Genes (DEGs)

Insights about the involvement of the different *PIN* family members on olive plant response to environmental stresses were retrieved from the analysis of paired end-reads from FastQ raw sequence data of an RNAseq experiment deposited in European Nucleotide Archive databases (https://www.ebi.ac.uk/ena (accessed on 20 October 2019)) identified with the accession number PRJNA256033 (https://www.ebi.ac.uk/ena/data/view/PRJNA256033&portal=read_experiment (accessed on 20 October 2019)). Cold stress in olive leaves and changes in gene expression on roots induced by wounding and *V. dahliae* infection were the considered stress factors. Briefly, total RNA was extracted from roots of control plants (uninoculated and unwounded), from micro-wounded roots at 2 and 7 days post wounding (dpw), and from *V. dahliae*-inoculated roots (isolate V937I) at the same timepoints (2 and 7 days post-inoculation, dpi). Regarding the study of transcriptional changes during cold acclimation in olive leaves, total RNA was extracted from leaves of unstressed plants (control) of cv. ‘Picual’ and from plants exposed to cold stress conditions (10 °C day/4 °C night) at 1 and 10 days after cold exposure (dac). Total RNA was used to synthetize cDNA libraries and sequenced using Illumina HiSeq 1000 platform. In each experiment, two replicates were considered per timepoint (see Supplementary Materials Table S2 for acc. numbers regarding each experimental condition/timepoint/replicate). For more detailed information regarding experimental design and procedure for cDNA libraries synthesis and sequencing, see [54,55], which first analyzed the full RNAseq data.

Retrieved sequences were trimmed using Trimmomatic [56] incorporated in the OmicsBox V2.1.2 software (BioBam Bioinformatics, Valencia, Spain) with default settings. Trimmed reads were imported into CLC Genomics Workbench 11.0.1 (Qiagen, Redwood City, USA) and mapped against the *O. europaea* cDNA RefSeq retrieved from http://denovo.cnag.cat/ (accessed on 30 November 2019) and containing 89.982 sequences [57]. The settings used were length fraction = 0.8, similarity fraction = 0.8, mismatch penalties = 2 and gap penalties = 3, as set by CLC default parameters. Counts were normalized using the TMM method. A CPM filter of 1 and a false discovery rate (FDR) < 0.05 were used to find differentially expressed genes (DEGs), through a General Linear Model (GLM) analysis (Likelihood ratio test) using treatments (cold/wounding/infection with *V. dahliae*) and time as factors.

The Gene Ontology (GO) enrichment analysis of the differentially expressed *OePINs* was performed using OmicsBox v2.0.36. software (BioBam Bioinformatics, Valencia, Spain) [58]. KEGG pathways assignment [59] was further performed using OmicsBox.

### 2.5. Construction of Gene Co-Expression Networks

An olive tree gene co-expression network related to wounding, infection with *V. dahliae*, or cold, was constructed using the WGCNA package [60] within the R environment. The default WGCNA “step-by-step network construction” analysis was used to build modules (clusters of genes displaying similar correlated patterns of transcription). The parameters used were soft-power of 12, 18, and 24 (for the experiment wounding, infection with *V. dahliae* and cold, respectively) and a minimum module size of 30 genes. The adjacency between genes was calculated and a hierarchical clustering tree with the dissimilarity of the topological overlap matrix was constructed. Similar modules were merged by calculating the module eigengenes, clustering them, and assigning a distance threshold. To identify modules that were significantly associated with wounding, *V. dahliae* infection and cold (either up- or down-regulated), the module eigengene was calculated for each module and then correlated with the treatment (α = 0.05). The *OePIN* genes were searched amongst the significantly correlated modules.

GO analysis was performed for modules containing the *OePIN* genes following the procedure described above. The KEGG Orthology database [59] was used to perform a further functional classification and pathway assignment of the up- and down-regulated DEGs, using OmicsBox.

## 3. Results

### 3.1. Identification of OePIN Genes—Chromosomal Distribution, Gene Structure and Phylogeny Analysis

Twenty-one *OePIN*-coding loci were identified by searching the genome database of *O. europaea* subsp. *europaea* var. *europaea* (cv. ‘Farga’, Oe6 browser), including two truncated loci (OE6A045718P1 located at the Oe6_s10141 scaffold and OE6A004471P1 located at the Oe6_s00066) (Table 1). Both sequences were truncated due to the existence of repetitive sequences or transposable elements (TEs). In the case of OE6A045718P1, the sequence was truncated at the end of the first exon due to an expanded tandem repeat region (the repeat motif was ca. 85-nt-long), resulting in a premature stop codon (not shown). In the case of locus OE6A004471P1, the sequence of intron 1 was disrupted by insertion of a gypsy-LTR element (not shown). Considering that both sequences likely encoded non-functional proteins, they were considered pseudogenes and were not considered further in in silico protein sequence analyses.

Besides truncated sequences, gene duplication events identified by high levels of sequence similarity were also identified. The loci OE6A036288P1/OE6A046725P1 and OE6A040519P1/OE6A094847P1 showed sequence similarity higher than 99% for both coding DNA and translated peptide sequences. Slight differences were identified at the genomic sequence level (including intron regions) due to variability at a few nucleotide positions (single nucleotide polymorphisms, SNPs) and short Insertion/Deletion (InDel) events, the latter responsible for minor variation in gene length (less than 20 bp). Such a level of sequence similarity led us to consider that both pairs of loci might have evolved from a single locus as a result of recent duplication events, here considered as single genes with two copies in the genome. From the 21 loci initially identified, a total of 17 members were considered as the composition of the *PIN* gene family of var. *europaea*. In var. *sylvestris*, from the initial 19 retrieved sequences, four were truncated (XM_023030314 located at NC_036237, XM_023033699 located at NC_036247, XM_023005297.1 located at NW_019241282.1, and XM_023039423.1 located at NC_036250) putatively leading to non-functional proteins (Table 2). Moreover, XM_022997346.1 located at NC_036238 was not included in the analysis, as the sequence was not contiguous. Therefore, from the nineteen sequences identified it were considered a total of 14 encoding functional proteins. Due to the high level of similarity among sequences from both varieties, all sequences were considered for phylogenetic studies to better understand gene radiation differences between both varieties, as well as to understand the dynamics of gene loss/gain.

Several events of gene duplication, confirmed by 100% similarity at the genomic sequence level, were also identified in var. *sylvestris*. One of those appeared on chromosome 1 (NC_036237) as a tandem duplication (XM_022996510 and XM_022996594), and another located on chromosome 19 (NC_036255) (sequences XM_022989965 and XM_022989968, also showing 100% similarity). This way, by discarding the sequences putatively encoding non-functional proteins and the duplicated sequences, the total number of genes that comprise the *PIN* gene family in var. *sylvestris* is 12 genes compared to the 17 considered in var. *europaea*.

Identification of gene locations by searching the information available for the var. *sylvestris* whole genome sequence revealed that *PIN* members were not evenly distributed on the chromosomes (Table 2). On chromosomes 2, 3, 4, 5, 7, 9, 13, 14, 15, 16, and 18 no *PIN* sequences were identified, while chromosomes 6, 8, 10, 11, and 17 carried one *PIN* sequence, chromosomes 12 and 19 comprised two *PIN* sequences, and three different *PIN* sequences were identified on chromosome 1.

**Figure 1:**
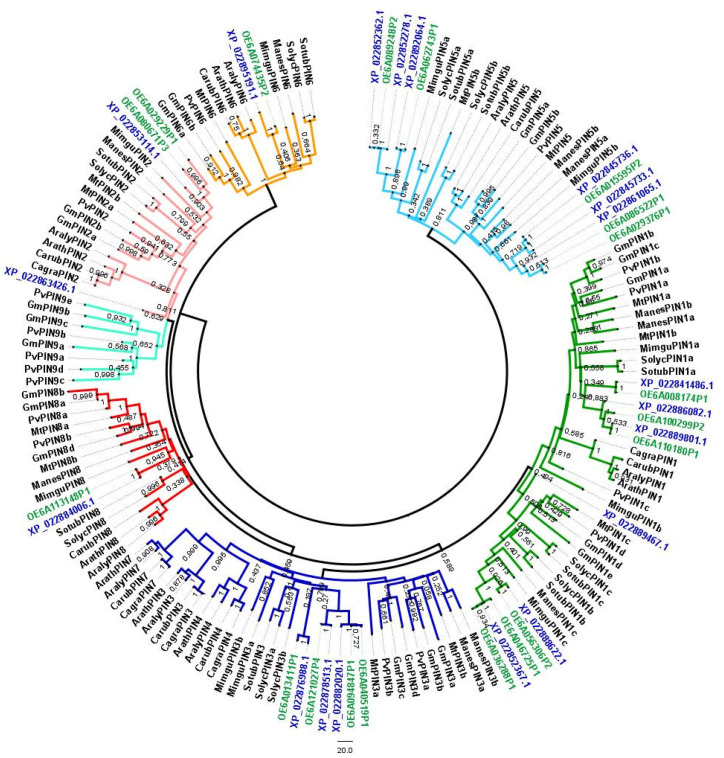
Neighbor-Joining (NJ) tree showing the phylogenetic relationships among deduced PIN sequences from 13 eudicot plant species. Deduced peptide sequences corresponding to both varieties of *Olea europaea* subsp. *europaea*, the var. *europaea*, and the var. *sylvestris*, were included (in green are shown sequences from var. *europaea* and in blue from var. *sylvestris*). 158 putative PIN sequences from higher plants were included (correspondence to accession numbers and the plant species is included in Supplementary Materials Table S1). The NJ tree was obtained using the complete peptide sequences. The alignments were bootstrapped with 1000 replicates by the NJ method using the MEGA 7 software. The scale bar indicates the relative amount of change along branches. PIN proteins can be divided into seven main branches, which correspond to the different subfamilies, here differentially colored.

Aiming to investigate the phylogenetic relationships among PINs from olive, a phylogenetic tree was built with 120 complete deduced peptide sequences retrieved from 11 plant species belonging to *Fabales*, *Brassicales*, *Solanales*, *Malpighiales*, and *Lamiales* (Figure 1). Protein sequences from *A. thaliana*, *A. lyrate* (L.) O’Kane & Al-Shehbaz, *G. max*, *Mimulus guttatus* Fisch. Ex DC., *Solanum lycopersicum* L., *Medicago truncatula* Gaertn., and *Phaseolus vulgaris* L. were retrieved from previous *PIN* family characterization reports [29,53]. Sequences from *Capsella grandiflora* (Fauché & Chaub.) Boiss., *C. rubella* Reut., *Manihot esculenta* Crantz, and *S. tuberosum* L. were retrieved from Phytozome v.12 (see details in Supplementary Materials Table S1). The newly identified genes were named according to the *PIN* subfamily to which they clustered, in accordance with the members already classified from the known species. The analysis revealed that these PINs could be divided into seven groups (subfamilies): PIN1, PIN2, PIN3/4/7, PIN5, PIN6, PIN8, and PIN9. Clusters of PIN4 and PIN7 only included members from Brassicaceae and clusters of PIN9 only included members from Fabaceae.

Compared to species that show no more than ten genes, which belong to *Brasicales*, *Solanales*, and the single *Lamiales* species (*M. guttatus*), *O. europaea* subsp. *europaea PIN* gene family, with 12 and 17 members considering var. *sylvestris* and var. *europaea*, respectively, is extensively expanded, more similar in terms of the *PIN* family size to *Fabales* that comprise 16 (*P. vulgaris*) and 21 (*G. max*) gene members (Figure 1, see details in Supplementary Materials Table S1).

*OePINs* can be grouped into long and short *PIN* sequences according to the predicted length of the encoded protein. The *OePIN* gene family from *O. europaea* var. *europaea* is composed of 11 long *PIN* members showing open reading frames (ORF) ranging from 1506 to 1929 bp (encoding putative peptides that range from 502–643 aa) and six short *PIN* members with ORF size of 1062–1149 bp (encoding peptides of 354–372 aa). *PINs* from *O. europaea* var. *sylvestris* present slight differences compared to var. *europaea* showing the loss of members for both long and short sequences, leading to eight long and four short *PINs* if considered the twelve sequences encoding functional PINs. Regarding the encoded peptides, there were similarities in terms of sequence length between both varieties (for details, see Table 1 and Table 2).

Besides the differences in the ORF sequence length that distinguish both long and short *PIN* genes, differences in the gene structure can also be pointed out. Typically, long *PIN* gene sequences comprise six exons interrupted by five introns, while short *PINs* show a structure with five exons and four introns (see details in Supplementary Materials Table S3). Genes sharing this structure usually present exon size conservation for the last four exons (86, 158, 77, and 67/73 bp, respectively). This characteristic is responsible for a similar protein size encoded by *PINs* across plant species. Size variability of *PINs* encoded by long/short genes with a conserved exon structure is mainly associated with variability of exons 1 and 2, although exon size can also vary in the last four exons due to events of exon loss or gain. This is the case of *OePIN8* from both *Olea* varieties in which exon 3 (244 bp-long) is the result of a fusion of exons 3 and 4 (86 plus 158 bp), consequently leading to an intron loss event.

Events of exon gain can also be identified in *OePINs*. The *OePIN6* member from var. *europaea* and the *OePIN2b* from var. *sylvestris* comprises seven exons. In the case of *OePIN6*, which shows conservation at the four last exons, it seems that exon 1 is split into three shorter exons. In the case of *OePIN2b*, the high variability in terms of the exon size results from an LTR-RT insertion described below.

In opposition to the high level of conservation observable at the protein-coding sequence across different *PIN* gene family members, introns present a high level of variability even when compared among long or short sequences within the same variety or across members from different varieties. Variability of the intron size can range from very short introns that go from 66 bp (intron 2 of *OePIN8* from both var. *europaea* and *sylvestris*) to very large introns with 2492 (intron 2 of *OePIN6* in both varieties) or even with 6370 bp, seen at intron 3 of *OePIN2b* from var. *europaea*.

**Figure 2:**
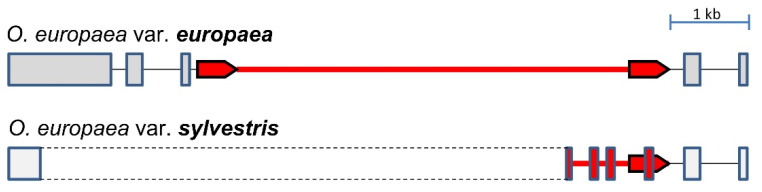
Schematic representation of the organization of the *PIN2b* gene in *Olea europaea* subsp. *europaea* var. *europaea* and *Olea europaea* subsp. *europaea* var. *sylvestris*. Boxes represent exons, dashed lines show deletion resulting in the formation of a truncated and rearranged exon 1 in var. *sylvestris* with the 3′ end originating from the copia-LTR retrotransposon, the red line represents the insertion of the copia-LTR retrotransposon, and red arrows show localization of its long terminal repeats (LTRs). The insertion is flanked by a 5-nt-long target site duplication (TSD: ATTCT, not shown). Red boxes in var. *sylvestris* show exons originating from the exapted copia-LTR fragment. The scheme is drawn to scale, bar = 1 kb.

The large intron size of *the OePIN2b* gene from var. *europaea* in addition to the non-conserved exon structure in the orthologous gene of var. *sylvestris* has led us to investigate in more detail the sequence composition of this gene in both genotypes. In the case of var. *europaea*, it carries an insertion of an LTR retrotransposon belonging to the copia-LTR superfamily, located between positions 2393 and 8438 bp (with left LTR of 526 bp located at position 2393–2918 bp, and right LTR of 535 bp located at position 7904–8438 bp, see details on Figure 2), exhibiting the polyprotein-encoding ORF oriented opposite to the reference strand. Judging from the similarity of the two LTRs, it is an old insertion, with an estimated date of insertion of 24.29 Mya, with numerous SNPs and one InDel event. Nevertheless, the TSDs (ATTCT or AGAAT for reverse complement) have been retained. The insertion of the copia-LTR retrotransposon in the homologous *PIN2b* member of *O. sylvestris* was followed by a partial deletion encompassing a 3′ portion of exon 1 and a large fragment of the retrotransposon. It resulted in the formation of a rearranged protein-coding sequence harboring new exons from the remaining portion of the retrotransposon (see details in Figure 2). That new arrangement at *PIN2b* member might have implications in terms of the protein functionality due to the lack of conserved domains (Mem-trans) at the protein C-terminus (see the transmembrane topology of var. *sylvestris* in Supplementary Materials Figure S1).

### 3.2. Protein Sequence Analysis

Analysis of protein sequences was performed considering only PINs retrieved from *O. europaea* var. *europaea*. According to the result achieved by searching for conserved domains within the 17 retrieved protein sequences (https://www.ncbi.nlm.nih.gov/Structure/cdd/wrpsb.cgi?RID=ACAK7J2A01R&mode=all (accessed on 30 July 2019)), two conserved domains, typical of members belonging to the superfamily of membrane transport proteins (Mem_trans, pfam03547), were identified across all OePIN sequences. A putative highly conserved hydrophobicity profile was shared among all sequences as all of them possessed, as conserved domains located at their N- and C-termini, hydrophobic segments composed of several transmembrane helices (each one with 18–23 amino acids in length). A central hydrophilic loop that links hydrophobic segments at N and C-termini can also be seen across all OePIN proteins. In var. *europaea* 11 members show large sequence sizes (ranging from 528 to 643 aa), including all members of the OePIN1, OePIN2, OePIN3, and OePIN6 subfamilies; while the typical short OePINs comprise six members, which belong to the OePIN5 and OePIN8 subfamilies (354–368 aa) (see detailed information at Supplementary Materials Table S3).

**Figure 3:**
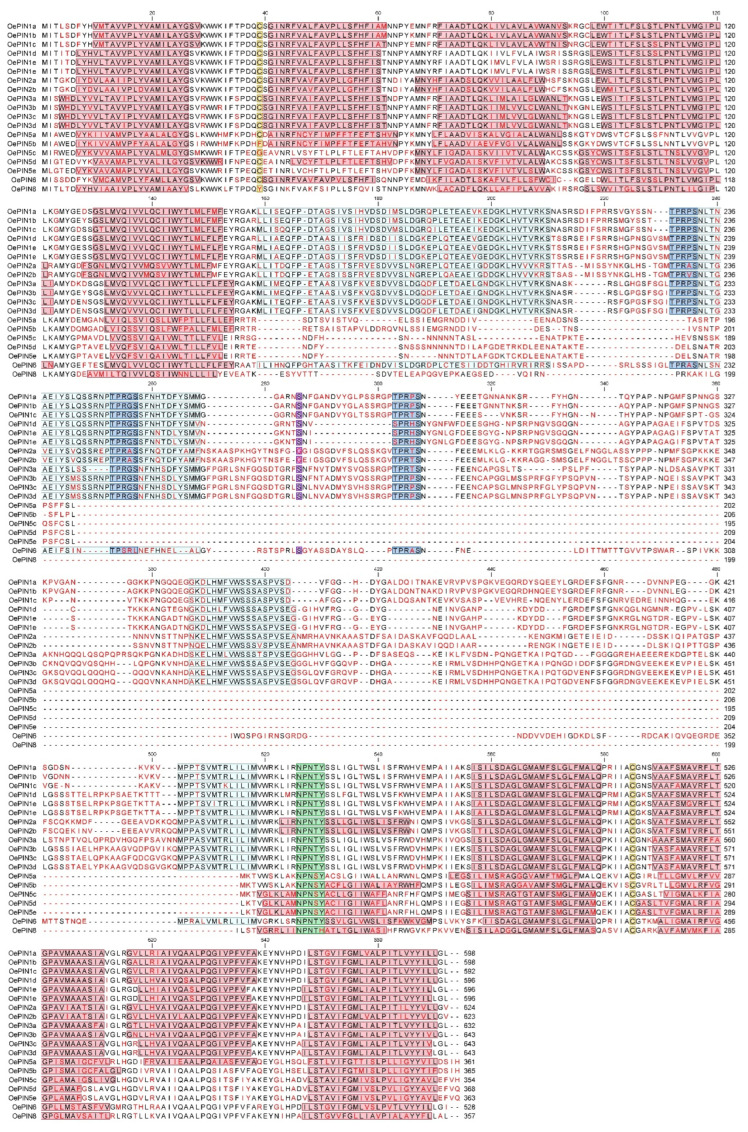
Multiple alignments of deduced OePIN sequences (Olea *europaea* subsp. *europaea* var. *europaea*) and identification of the principal protein domains already described in other plant species. Amino acids block colored in red represent predicted transmembrane helices, separated by loop domains (based on in silico analysis using the THMM automatic server freely available at http://www.cbs.dtu.dk/services/TMHMM-2.0/ (accessed on 30 July 2019)). The conserved HC1-HC4 regions within the central loop domain are highlighted in grey. Conserved cysteines located at both transmembrane domains, previously identified in AtPIN (Cys-39 and Cys-560) as cis-acting regulators of protein polar localization, are displayed in yellow. In blue are shown the conserved TPRXS (N/S) motif across canonical sequences that work as targets of the PINOD (PID) family kinases, D6 protein kinase (D6PK), and mitogen-activated protein kinase (MAPK) for phosphorylation of the serine or threonine residues. The position of one additional serine residue (S4), typically conserved across PIN sequences, is highlighted in purple. The internalization motif NPXXY is shown in green.

To investigate the conservation of the HC1–HC2 regions, as well as the principal protein structural features already described in other plant species, a multiple sequence alignment was done using the 19 complete sequences retrieved from the olive genome website (*O. europaea* subsp. *europaea* var. *europaea* cv. ‘Farga’) which encode 17 *PIN* members. Figure 3 summarizes the predicted structural features identified across OePIN sequences. In terms of the sequence composition, a high level of conservation is shown at the N- and C-terminal transmembrane segments across all OePIN protein members. Contrarily, an overall lack of conservation in the center of the protein sequences, which corresponds to the hydrophilic loop sequences, resulted in a generally poor alignment. Conservation of HC1–HC2 motifs is clearly seen for the OePIN1, OePIN2, and OePIN3 subfamily members, which allows us to classify these members as canonical PIN proteins. Contrarily, all members of OePIN5 and OePIN8 completely lack this region or fail in terms of sequence homology, which leads us to consider these members as non-canonical PIN proteins. Curiously, OePIN6 appears like an intermediate member showing conservation at the HC1 and HC4 motifs but very poor homology at the HC2 and HC3 motif sequences.

PIN members belonging to the canonical PINs present a central hydrophilic loop that ranges from 303–362 amino acids in length, while non-canonical members present shorter sequences from 46–85 amino acids (Figure 3). Besides the HC motifs located at the hydrophilic loop, some additional motifs were identified at the central protein region conserved across canonical sequences. Three conserved TPRXS (N/S) motifs were identified; however, some amino acid changes can be seen. OePIN1d and OePIN1e show SPRHS, and OePIN6 show TPSRL.

Predicted transmembrane helices that compose the N- and C-termini of each sequence are highlighted in Supplementary Materials Table S4, making visible the variability in terms of the number of helices that compose each hydrophobic segment (see transmembrane prediction results of var. *europaea* in Supplementary Materials Figure S2). Typically, non-canonical OePIN proteins harbor eight transmembrane helices (exception of OePIN5d), while canonical OePIN proteins harbor nine to ten transmembrane helices (exceptions seen in OePIN1d and OePIN1e showing only eight). The presence of the internalization motif NPXXY [61], located at the beginning of the C-terminal hydrophobic domain, is displayed. The conserved tyrosine (Y) is present across all OePIN members with the exception of OePIN8 which carries a histidine (H) instead (Figure 3).

Two conserved cysteines, previously identified in AtPIN at positions Cys-39 and Cys-560 [62], were also identified and highlighted with few exceptions identified at non-canonical PINs (OePIN5b, OePIN5c, and OePIN8 are lacking Cys-39). Both Cys are located at the loop sequence, the first one located at the first hydrophobic segment and the other one at the second hydrophobic segment. In canonical sequences and most of the non-canonical ones, Cys-39 residue lies in the junction between helix 1 and 2, at loop 1 (OePIN5e is the exception), whereas Cys-560 is located, for most of the canonical sequences (exceptions are both OePIN2 and the single OePIN6 member), between helices 6 and 7, at loop 6. Considering topology predictions, the central loop region of OePINs would face the intercellular environment, which indicates that Cys-39 and Cys-560 would be localized at the cytoplasmic side of the plasma membrane [63]. Prediction of the subcellular localization of OePIN proteins was also carried out, and the results from the GO categorization related to cellular component revealed a localization in multiple cellular components, which includes endoplasmatic reticulum (17), cell wall/membrane (38), plasmodesma (11), making part of cell composition (7) or being localized on its surface (4), or even being part of vesicle and vacuole membrane (4 and 1, respectively) (see Supplementary Materials Figure S3b). Complementary analysis performed at PSORT using each OePIN translated sequence revealed a predicted localization in the plasma membrane of all canonical members, with an exception seen for OePIN2b, while non-canonical ones were mainly predicted to be located at the endoplasmic reticulum (see Supplementary Materials Table S4). Putative signal peptide shows high conservation across PIN gene members (Supplementary Materials Table S4).

### 3.3. High-Throughput Expression Analysis of OePINs

RNA-Seq data retrieved from the European Nucleotide Archive database (https://www.ebi.ac.uk/ena/data (accessed on 20 October 2019)) were used to investigate *OePIN* expression profile upon two abiotic stress factors (cold and wounding) and during plant infection with the pathogenic fungus *V. dahliae*, in *O. europaea* L. cv. ‘Picual’ plants. A total of 89,982 sequences included in the olive reference transcriptome (http://denovo.cnag.cat/olive (accessed on 30 November 2017), Oe6 browser) were used for mapping. The number of assembled transcripts retained after CPM filtering used for subsequent analysis searching for the existence of DEGs ranged from 40,213 to 45,532 (shown in Supplementary Materials Table S5).

The putative involvement of *PIN* genes in plant response to stress was firstly evaluated by categorization of *OePIN* transcripts into GO classes (Biological Process (BP), Cellular Component (CC), and Molecular Function (MF)) (Supplementary Materials Figure S3) to further compare with data achieved when considered the total of DEGs. BP terms were mostly related to “auxin homeostasis”, “auxin efflux” and “transmembrane transport” (Supplementary Materials Figure S3a). The CC and MF classes were also enriched in auxin-related terms: “auxin efflux carrier complex”, “efflux transmembrane transporter activity”, “auxin transmembrane transporter activity” (Supplementary Materials Figure S3b,c).

Regarding the total of DEGs, the ontology terms (Biological Process class) identified in both up- and down-regulated genes at 2 and 7 days post-wounding (dpw) were mostly related to “transport/transmembrane transport” (Supplementary Materials Figure S4). The terms “response to stimulus” and “oxidation-reduction process” were also highly enriched in upregulated genes (2 dpw and 7 dpw) and to a less extent in down-regulated genes at 7 dpw.

The BP terms identified in upregulated genes at 2 and 7 days post infection (dpi) with *V. dahliae* were “cellular protein modification process”, “oxidation-reduction process”, “transport,” and “phosphorylation” (Supplementary Materials Figure S5). The term “response to stimulus” was only enriched at 7 dpi. Regarding the down-regulated genes, “RNA metabolic process” and “transmembrane process” were highly enriched at 2 dpi and to a less extent at 7 dpi (Supplementary Materials Figure S5). The processes “cellular macromolecule biosynthetic process” and “gene expression” were also common to both time points.

The cold stress also affected the Gene Ontology of DEGs at 1 and 10 days after cold (dac) (Supplementary Materials Figure S6). Interestingly, the term “transport” was highly enriched in the upregulated genes, particularly at 1 dac. At 1 dac “response to stimulus” and “regulation of transcription, DNA-templated” comprised 36% of the sequences and were not found at 10 dac. For the down-regulated genes, the terms “gene expression,” “cellular protein modification process,” and “cellular macromolecule biosynthetic process” were found at 1 and 10 dac, amongst others (Supplementary Materials Figure S6).

**Figure 4:**
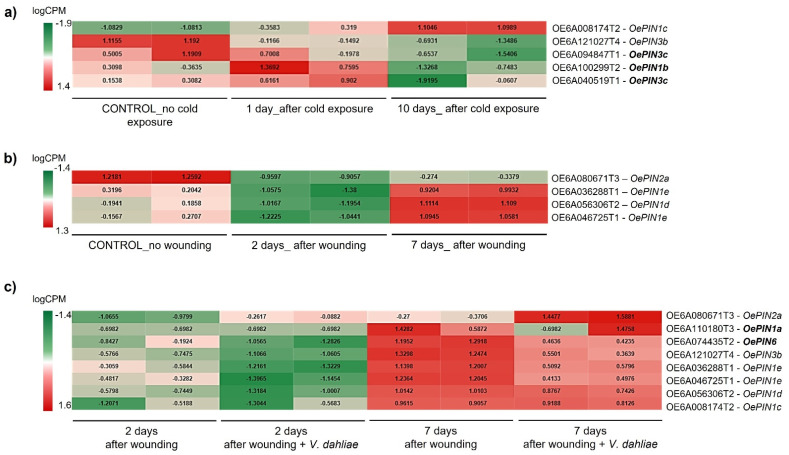
Heatmaps showing differentially expressed *OePIN* genes in the three different conditions evaluated: (**a**) exposure to cold stress, (**b**) wounding, and (**c**) infection with *Verticillium dahliae*. A false discovery rate (FDR) <0.05 was used to find differentially expressed genes through a General Linear Model (GLM) analysis (Likelihood ratio test) using treatments (cold/wounding/infection with *V. dahliae*) and time as factors. In bold there are transcripts associated to a single stress condition.

**Figure 5:**
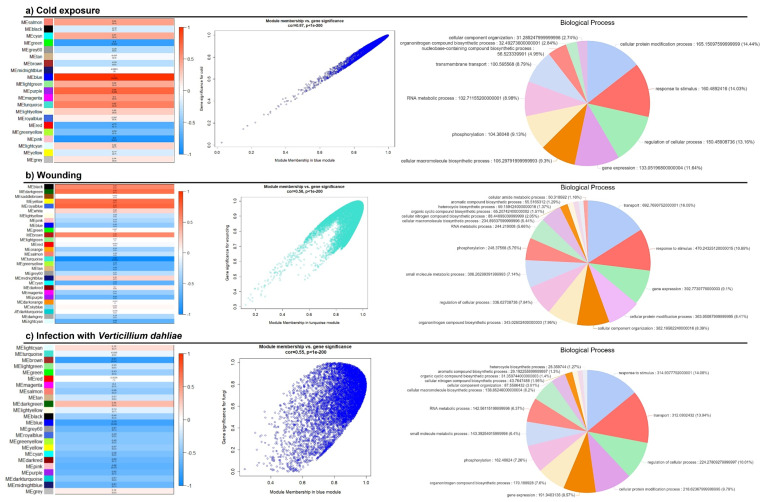
Modules (clusters of genes displaying similar correlated patterns of transcription) identified through a gene co-expression network constructed using the WGCNA package related to cold exposure (**a**), wounding (**b**), and infection with *Verticillium dahliae* (**c**). Genes belonging to each selected module were grouped according to the biological process they are involved in (pie-charts presented for each condition), achieved through a GO enrichment analysis within OmicsBox software.

From screening of DEGs in stress conditions, different transcripts encoding canonical PIN proteins were identified across the three stresses, which included all members of *OePIN1*, the *OePIN2a* member, and some *OePIN3* members (*OePIN3b* and *OePIN3c*) (see Figure 4a and Figure 5b,c). Contrarily, members encoding non-canonical PINs were mostly absent across all conditions; of those, only a single member of *OePIN6* was identified (see Figure 4c). Members of *OePIN5* and *OePIN8* were not detected within DEGs in any of the tested stresses.

The data clearly showed a link between the differentially expressed *OePIN* members and stress conditions (Figure 4). Upon exposure to cold, only members belonging to subfamilies *OePIN1* and *OePIN3* were identified as DEGs. From those, three genes appear as cold stress-specific (*OePIN1b*—OE6A100299T2, and both *OePIN3c*—OE6A094847T1 and OE6A040519T1). Regarding the expression pattern, both *OePIN1* members (*OePIN1b* and *OePIN1c*) and the *OePIN3c* (OE6A040519T1) were upregulated 1 dac while *OePIN3b* and *OePIN3c* (OE6A094847T1) were downregulated. After a long period of cold exposure (10 dac), most of the *OePINs* were down-regulated. The only exception was *OePIN1c*, exhibiting upregulation at 10 dac.

It is interesting to note that instead of the principal isoform (identified as T1) many alternative transcripts, encoding for smaller peptides, were detected within the DEGs. There are examples of an alternative isoform 2 for *OePIN1b* and *OePIN1c*, and an alternative isoform 4 identified in the case of *OePIN3b*. The identification of alternative transcripts within the DEGs is not exclusive to cold stress, as they can be identified across all three stress conditions (see Figure 4a–c).

Regarding the olive response to wounding, four *OePINs* were identified. Amongst the studied stress conditions, wounding presented the lowest number of differentially expressed *OePINs*. The four genes identified, even identified in association with *V. dahliae* as well, were not detected as cold-stress responsive. Overall, all wound-responsive *OePINs* were down-regulated 2 dpw and later, at 7 dpw, appeared as upregulated. From those, the *OePIN1* members were the genes exhibiting a higher transcript accumulation.

Regarding plant response to both wounding and fungal infection, the majority of the *OePIN* genes were downregulated 2 days after stress exposure (either after wounding or fungal infection), albeit more severely following inoculation with *V. dahliae*, while on the seventh day (7 dpw or 7 dpi), the genes were upregulated (Figure 4c). *OePIN1a* and *OePIN6* were only found to be differentially expressed when data from wounding and fungal infection was analyzed simultaneously and showed similar expression patterns, being up-regulated at 7 dpi (Figure 4c). Amongst all *OePINs*, *OePIN2* appears as the most upregulated one at 7 dpi, indicating a putative role in fungal infection response.

### 3.4. Weighted Gene Co-Expression Network Analysis (WGCNA)

Aiming to get insight into a biological network involving *PIN* genes, a WGCNA analysis was performed, and further identification of *PIN* genes on significantly correlated modules was performed. For each experiment, a dynamic hierarchical tree algorithm was used to divide the clustering tree, resulting in 29, 24, and 21 expression modules for wounding, *V. dahliae* infection, and cold, respectively. Gene co-expression modules showed that module “turquoise” was tightly correlated with wounding (down-regulated) (*p* < 0.001), module “blue” was tightly correlated with fungal infection (down-regulated) (*p* < 0.001) and module “blue” was correlated with cold (upregulated) (*p* < 0.001) (Figure 5). A large number of transcripts were identified belonging to each of the selected modules, with 14,263, 10,950, and 8094 for wounding, fungal infection, and cold, respectively. GO enrichment analysis of all genes showed that “turquoise—wounding” was enriched in terms, such as “transport,” “response to stimulus” and “gene expression” (Figure 5a); in the “blue—*V. dahliae*” the “regulation of cellular process” and “gene expression” were the most enriched terms (Figure 5b), and in “blue—cold” the top GO terms were “cellular protein modification process”, “response to stimulus,” and “regulation of cellular process” (Figure 5c).

KEGG pathway mapping revealed that the number and the pattern of activated pathways were similar across the three stress conditions (Supplementary Materials Figure S7). Of those, the “environmental information processing” was the highest activated pathway (>20% seqs), which corresponded to “signal transduction.” Looking at the genes grouped in each module, a high number of auxin-related genes were identified, including signaling, metabolism, and transport pathways. A total of 93 auxin-related genes were identified as wounding-related, 78 in response to *V. dahliae*, and 61 cold-responsive. A comparison of the differentially expressed auxin-related genes (63 specific to wounding, 50 specific to fungal infection, and 51 specific to cold) revealed that only a few transcripts were common to the different stress factors, including members of AUXIN SIGNALING F-BOX 2-like, auxin-responsive SAUR-like, auxin-responsive IAA-like, auxin response factor (*ARF*)-like, ABC transporter B (*ABCB*) and auxin transporter-like protein (*LAX*). Differences among the members of each gene family were identified across each stress factor (not shown). Additionally, *YUCCA10* (indole-3-pyruvate monooxygenase, involved in auxin biosynthesis pathway) and *YUCCA3* were detected in wounding and *V. dahliae* hub, respectively, and no *YUCCA* genes were detected upon cold stress.

*OePIN* genes were found within each one of the significantly correlated modules. *OePIN3b* (OE6A121027T4) was found in the “turquoise” module related to wounding, *OePIN1a* (OE6A110180T1) and *OePIN2a* (OE6A080671T3) were found in the “blue” module related to fungal infection, and *OePIN1a* (OE6A110180T4), *OePIN1b* (OE6A029229T1), *OePIN3c* (OE6A094847T1), *OePIN5d* (OE6A086522T1), and *OePIN5e* (OE6A029376T1), were found in “blue” module related to cold exposure.

A high number of genes related to Ca^2+^ sensing (encoding calmodulin, CaM, and calcineurin B-like protein, CBL) and transport pathways (Ca^2+^-ATPases and H^+^/Ca^2+^ antiporters) were identified across the three hubs. Additionally, genes encoding enzymes involved in the homeostasis of reactive oxygen species (ROS), used by plants as stress signaling molecules, were identified in each hub, with higher numbers in wounding and fungal infection. These were superoxide dismutase [Cu/Zn/Mn/Fe-*SOD*], catalase (*CAT*), and members of ascorbate peroxidase (*APX*), glutathione reductase, glutathione S-transferase, glutathione peroxidase, and alternative oxidase (*AOX*) families.

## 4. Discussion

### 4.1. OePIN Gene Family Reveals High Diversity and a Genotype-Specific Radiation Pattern

PIN proteins are auxin export carriers that direct intercellular auxin flows, regulating diverse aspects of plant growth and development, including responses to environmental constraints. In olive, the first reference to the *PIN* gene family has been made by Velada and co-authors [45], showing its involvement in response to wounding and to IBA in association with the development of adventitious rooting. In the present study, 17 putative *PIN* functional sequences in *O. europaea* var. *europaea* and 12 in var. *sylvestris* were identified. Differences involve not only the number of genes but also the presence of transposable elements that could be associated with differences in the genome evolution after duplication events. In olive, the occurrence of three differentiated waves of massive gene duplications has been reported [63]. It is known that after an event of polyploidization, the genomes tend to return to the diploid state through events of chromosome fusion or loss, (retro)transposon mobility, repetitive DNA loss, and gene loss [64]. In each genotype, *OePIN* members belonging to the same subfamily and located at the same scaffold/chromosome could have evolved from the same gene ancestor after an event of genome duplication. Members of *OePIN2*-subfamily, located at Oe6_s09941 scaffold (*OePIN2a* and *OePIN2b*) and *OePIN5*- subfamily, located at Oe6_s02490 (*OePIN5c*, *OePIN5d*, and *OePIN5e*) are examples of genes that likely evolved from a common ancestor. Gene duplication events, implemented by segmental duplication and tandem duplication mechanisms, have accompanied the expansion of gene families and, consequently, the evolution of plant genomes [65]. Identification of gene duplication events led us to consider 12 gene members in var. *sylvestris* and 17 in *europaea*. Considering that var. *sylvestris* presents a genome size of 1.46 Gb and *europaea* ~1.3 Gb [66], no correlation was established between genome size and the number of *OePIN* gene family members. Yang and co-authors [67] highlighted that the diversity with respect to the *PIN* gene family composition and the gene family expansion was driven by plant exposure to environmental stresses that generated new functions resulting in more efficient plant plasticity.

Characterization of the *PIN* gene family was initially made by Paponov and co-authors [28] in *A. thaliana* and eight sub-families were identified. In the Neighbor-joining tree (Figure 1), a conserved phylogenetic structure across eudicot plant species, with members of different species clustering together, defines a subfamily composition. Usually, species more closely related present more similarity in terms of the *PIN* family composition (see the composition of the *PIN* gene family in the species used for phylogenetic analysis in Supplementary Materials Table S1) and subfamily radiation, which have been reported in different gene families [12,68,69]. It is also visible that only very closely-related species clustered together with members of AtPIN4 and AtPIN7. It has been suggested that homologous genes belonging to the same cluster, which in fact represent a subfamily, might share the same or overlapping functions [67].

Additionally, it is interesting to note that members of these two subfamilies belong to the PIN3 cluster, grouping members from all species included in the phylogenetic analysis. Following the overall evolution trend, the linage-specific expansion through partial genome modifications is characteristic of paralogous gene families, which according to several authors, was motivated by environmental pressures requiring an evolution through efficient adaptation [67].

### 4.2. Variability of OePIN Gene Structure Resulting from Mechanisms Acting at Genomic and Transcriptomic Level

OePINs carry a central hydrophilic domain within two highly conserved hydrophobic domains located at N- and C-termini, which exhibit high conservative positions according to the published model [53]. At the N-terminus of the proteins, most OePIN members present five trans-membrane domains (Helix 1–5), while at the C-terminus, most of the members are characterized by the presence of four trans-membrane domains. Exceptions have been reported, including the absence of helix2 at the N-terminus and helix7 at the C-terminus in PIN8 of *S. tuberosum* [67]. Similarly, highly conserved domains HC1–HC4 were identified in the majority of OePIN members (OePIN1, OePIN2, OePIN3, and the single member of OePIN6).

*PIN* gene structure has been explored in different reports showing high conservation across members of two main *PIN* groups [32], which is also characteristic of other plant gene families [68,69]. Genes encoding canonical PINs are characterized by a structure of 6 exons interrupted by 5 introns, while most of the genes encoding non-canonical proteins comprise 5 exons interrupted by four introns. Events of intron loss and gain, reported as one of the major driving forces for intron evolution [70], were identified in both genotypes, with a maximum of seven exons (seen in *OePIN6* of var. *europaea* and *OePIN2b* of var. *sylvestris*). Another characteristic of introns in *PIN* members is the high diversity of sequence length. Variability at intron sequences has been related to its functional role in the regulation of gene expression due to the possibility they offer to accommodate potential regulatory sequences [71]. Harbour of miRNA binding sites, encoding pre-miRNA sequences or transposable elements, and the existence of alternative polyadenylation sites leading to alternative polyadenylation events (APA) or alternative intron splicing (AS) are examples of mechanisms that could be involved in the control of gene expression [12]. APA allows a single gene sequence to encode diverse transcripts by changing the length of untranslated regions (UTRs) or coding regions [72] and depending on the location of the alternative poly(a) signal; gene expression could be affected qualitatively or quantitatively. The production of more than one mRNA may further lead to the translation of different protein isoforms with differences in cellular localization, stability, or function by changing or completely removing functional domains (alternative transcripts are identified in each *OePIN* member and the mechanism behind its origin are shown in Table 1 and Table 2). Widespread across all eukaryotic species, APA events have been highlighted as the major mechanism of gene regulation with consequences on physiological and biochemical processes [73]. Different reports have associated APA events with flowering regulation, growth and developmental processes, and response to stress [12,74]. Even if not explored at the functional level, it is curious to observe that most of the *OePINs* produce alternative transcripts showing significantly different expression patterns (see Figure 5). Further studies will be of high interest to understand the mechanism of APA associated with *OePINs* and its biological role in plant stress response.

Besides AS and APA events, epigenetic mechanisms have also been correlated with changes at the phenotype level, involving aspects of plant development and growth and also a response to stress conditions (see review in [75]). Transposable element insertions, known as mobile elements that selfishly replicate at the expense of host fitness, present a significant impact on genome evolution and on gene functioning. The present study allowed the identification of a copia-LTR retrotransposon at *OePIN2b* of var. *europaea*. The comparison between sequences of the two varieties revealed the exaptation of partial sequences of that element as exons of the *PIN2b* of var. *sylvestris*, which reveal such kind of event as a driving force in the olive *PIN* gene family evolution. The role of TE insertions in providing advantages upon environmental constraints has been highlighted (reviewed by [76]). In plants, TEs are strongly repressed by epigenetic silencing, mainly associated with high levels of DNA methylation and repressive histone modifications, but under certain circumstances, this repression is alleviated, allowing them to move, consequently rewiring new genes to the stress-related transcription networks, providing the plant with the capacity to adapt to new environments [77]. In addition to epigenetic events that take place before transcription and to changes that happen during transcription, reversible post-translational protein modifications have also been associated with the functional regulation of PIN proteins. In general, the addition to phosphorylation and ubiquitination of diverse amino acid residues in the protein chain, modifications of cysteines (Cys) play a decisive role in the post-translational control of proteins. This amino acid represents the principal target of Reactive Oxygen Species (ROS) [78] and a link between auxin and redox signaling has recently been established [79]. In the present report, OePIN showed complete conservation of Cys-560, while Cys-39 was not identified in OePIN5b, OePIN5c, and OePIN8. According to previous studies involving site-directed mutagenesis of one or both conserved Cys, it was demonstrated that protein mobility and localization in plasma membrane micro-domains could be affected [62]. Olive translated PIN sequences lacking Cys-39 correspond to non-canonical PINs, which commonly carry more than the two conserved Cys, (8 identified in OePIN6 and 5 identified in OePIN5a), which could replace the absent Cys in terms of their function.

### 4.3. OePINs Are Co-Expressed with Members Belonging to Diverse Stress Signaling Pathways and Oxidative Stress Homeostasis upon Biotic and Abiotic Stresses

Plant plasticity upon environmental constraints is influenced by reciprocal interactions between oxidative stress caused by ROS, auxin metabolism, long-distance transport, and directional cell-to-cell translocation [8]. Perturbations in auxin homeostasis can lead to adjustments in plant growth and development as a consequence of the activation of genes involved in plant plasticity by regulating cellular ROS levels through the action of an enzymatic scavenging system or by a non-enzymatic system involving the synthesis of antioxidants [80]. Moreover, Ca^2+^ has been described as the most important messenger in living plant cells exposed to biotic and abiotic stresses [81,82], with an important role in the activation of ROS synthesis, where H_2_O_2_ works as a long-distance signaling molecule [80]. Upon specific environmental stimuli, a transient increase in cytosolic [Ca^2+^], known as “calcium signature”, is generated [81,82]. Calmodulin (CaM), CML (CaM-like protein), CBL (calcineurin B-like protein), and CDPK (Ca^2+^-dependent protein kinase) are the proteins working as sensors of “calcium signature” [83,84]. To further restore the levels of cytosolic [Ca^2+^], the Ca^2+^ efflux into the cell exterior is sequestrated into cellular organelles such as vacuoles, endoplasmic reticulum, and mitochondria. The removal of Ca^2+^ from the cytosol against its electrochemical gradient requires energized active transport involving the Ca^2+^-ATPases and H^+^/Ca^2+^ antiporters. Genes encoding proteins involved in sensing the "calcium signature” and restoring [Ca^2+^] cyt homeostasis were identified in the three gene hubs, revealing a co-expression of these signaling pathways and *PIN* members in the three stress factors under study. Therefore, the sequestration of Ca^2+^ into the mitochondria could lead to the production of reactive oxygen species (ROS), which consequently activate oxidative stress signaling and simultaneously impact the auxin stability by oxidizing IAA via induction of peroxidase activity and redistribution through repression of the polar auxin transporters [83].

Different reports suggest that diverse biotic and abiotic stresses can modulate the differential auxin distribution through the regulation of PIN polar transport [84]. Auxin redistribution through down-regulation of different *PIN* members has been reported as a consequence of ROS production in different plant species upon diverse stress conditions. Structural differences that distinguish canonical and non-canonical PINs have conduced authors to hypothesize the association of proteins from each group to different functions. Usually, a similar function described in *Arabidopsis* is proposed for its ortholog in other plant species, and canonical PINs, located at the plasma membrane, have been indicated as responsible for cell-to-cell auxin polar transport. Down-regulation of *OePIN1*, *OePIN2*, and *OePIN3* members upon wounding and fungal infection suggests a reduction in auxin transport with a consequent effect on auxin redistribution as a consequence of ROS levels. Both wounding and fungal infection seem to be associated with the production of higher levels of ROS, explained by the higher number of genes encoding ROS scavenging enzymes [e.g., located at the mitochondrion: manganese superoxide dismutase (*MnSOD*), at the cytosol: ascorbate peroxidase (*APx*), cooper-zinc superoxide dismutase (*CuZnSOD*), and glutathione peroxidase (*GPx*); at the peroxisome: catalase (*CAT*)]. In addition, the interaction between *AOX* genes, involved in cellular ROS homeostasis and plant plasticity, and auxin has been highlighted [15]. Different reports show the identification of several auxin-responsive cis-acting regulatory elements located at the promoter region of *AOX1*-subfamily genes associated with abiotic plant stress response upon cold stress [12,85]. Members of the *AOX* gene family were also identified as co-expressed with *PIN* members upon cold stress, but a different expression pattern was observed in some *OePIN* members, which exhibited up-regulation at the early timepoint.

For *PIN* members encoding non-canonical proteins, known for their reduced central hydrophilic loop and location at the ER, only *OePIN6* was detected and associated with plant response upon fungal infection. *OePIN6* as a member encoding of non-canonical PINs would putatively be involved in intracellular regulation of auxin homeostasis [29]. Considering that most of the identified *OePIN* genes encode canonical PINs the hypothesis can be raised, highlighting the role of polar transport as a common mechanism involved in plant stress response to wounding, fungal infection, and cold exposure.

The involvement of auxins in plant resistance to different pathogens has been demonstrated, and the down-regulation of the auxin response upon *V. dahliae* has been correlated with the toxic effects of phytotoxins and other molecules secreted for degradation of plant cell tissues [86]. *Verticilium* wilt represents one of the most nefarious pathogens of olive cultivation, causing high economic losses due to the high susceptibility of olive cultivars. This pathogen infects the plant through the roots, and once in the vascular system, it spreads throughout the entire plant [87]. Plant pathogens can manipulate auxin biosynthesis, signaling, and transport pathways, promoting host susceptibility [88]. Crosstalk between the major two plant signaling pathways that mediate pathogen defense has been reported. A synergistic interaction between the jasmonic pathway explains the reduction of *PIN1* and *PIN2* auxin transporters in response to high JA accumulation [89]. Contrarily, the antagonistic crosstalk between auxin and salicylic acid (SA) leads one to hypothesize that the down-regulation of auxin signaling works as a part of the SA-mediated disease-resistance mechanism [88].

## 5. Conclusions

The present research, focused on in silico approaches, provides a deep characterization of the *PIN*-formed gene family in *O. europaea* L., providing information about the gene family characterization and expression patterns of the olive *PIN* members upon different stress conditions. The results provide important insights into the involvement of different *OePIN* members in plant responses to wounding, infection with *V. dahliae*, and cold exposure that could be taken into consideration for future experiments focused on functional analysis.

## Figures and Tables

**Table 1:** Identification of *PIN* gene family members from *Olea europaea* subsp. *europaea* var. *europaea*. Sequences were retrieved at the olive whole genome project website (cv. ‘Farga’, http://denovo.cnag.cat/genomes/olive/ (accessed on 30 November 2017)). In bold, there are the transcripts that encode the protein complete sequence.

Gene	Locus ID	Gene (bp)	Orf (bp)	Event
*PIN1* subfamily				
*OePIN1a*	**OE6A110180P1**	3287	1794	-
	OE6A110180P2		1731	APA_Int5
	OE6A110180P3		1677	APA_Int4
	OE6A110180P4		1530	APA_Int3
*OePIN1b*	**OE6A100299P2**	3818	1794	-
*OePIN1c*	**OE6A008174P1**	3192	1776	-
	OE6A008174P2		1707	APA_Int4
*OePIN1d*	**OE6A056306P2**	2853	1788	-
	OE6A056306P3		1404	APA_Int2
*OePIN1e*	**OE6A046725P1**	2746	1788	-
*OePIN1e*	**OE6A036288P1**	2740	1788	-
*	OE6A045718P1	1681	858	-
*	OE6A004471P1	1191	1188	-
*PIN2* subfamily				
*OePIN2a*	OE6A080671P1		1557	APA_Int2
	OE6A080671P2		1685	APA_Int3
	**OE6A080671P3**	3332	1872	-
*OePIN2b*	**OE6A029229P1**	9225	1869	-
*PIN3* subfamily				
*OePIN3a*	**OE6A013411P1**	2873	1896	-
	OE6A013411P2		1866	AS_Int1
*OePIN3b*	OE6A121027P3		1866	APA_Int5 + AS_Int4
	**OE6A121027P4**	2927	1929	-
*OePIN3c*	**OE6A040519P1**	2897	1929	-
	OE6A040519P2		1905	AS_Int1
*OePIN3c*	**OE6A094847P1**	2881	1929	-
	OE6A094847P2		1866	APA_Int5
	OE6A094847P3		1905	AS_Int1
*PIN5* subfamily				
*OePIN5a*	OE6A089248P1		1017	APA_Int3
	**OE6A089248P2**	1689	1149	-
*OePIN5b*	**OE6A062743P1**	1567	1095	-
*OePIN5c*	OE6A015595P1		1041	AS_Int1
	**OE6A015595P2**	1681	1062	-
	OE6A015595P3		1002	APA_Int3
	OE6A015595P5		1164	AS_Int2
*OePIN5d*	**OE6A086522P1**	1736	1104	-
*OePIN5e*	**OE6A029376P1**	1713	1089	-
*PIN6* subfamily				
*OePIN6*	OE6A074435P1		1506	APA_Ex7
	**OE6A074435P2**	5086	1584	
*PIN8* subfamily				
*OePIN8*	**OE6A113148P1**	2376	1071	-

* These sequences were truncated at 5′ or 3′-end and, for that reason, were considered pseudogenes; Event: corresponds to the event which gave rise to transcript variation; APA: Alternative PolyAdenylation; AS: Alternative Splicing.

**Table 2:** Identification of *PIN* gene family members from *Olea europaea* subsp. *europaea* var. *sylvestris*. Sequences were retrieved at the olive whole-genome project website (https://www.ncbi.nlm.nih.gov/genome/?term=Olea+europaea+var.+sylvestris+genome (accessed on 1 October 2018)).

Gene	Locus ID	Chr.	Position	Gene (bp)	ORF (bp)	Accession	Event
*PIN1* subfamily							
*OePIN1a*	NC_036248	12	3,293,149..3,297,089	3288	1794	X1: XM_023034033.1 X2: XM_023034034.1 X3: XM_023034035.1	-
					1794		-
				3308	1794		APA_int5+AS3′utr
***OePIN1b***	NC_036237	1	58,361..60,794	1697	1530	XM_023030314.1	
*OePIN1c*	NC_036253	17	15,003,301..15,007,290	3187	1776	X1: XM_022985718.1	
					1776	X2: XM_022985719.1	
*OePIN1d*	NC_036247	11	32,656,543..32,660,906	3639	1800	XM_023032854.1	
*OePIN1e*	NW_019229312	Unk	244,819..247,974	2741	1788	XM_022996599.1	
*	NC_036247	11	32,669,953..32,671,485	1212	597	XM_023033699.1	
*PIN2* subfamily							
***OePIN2a***	NC_036238	2	26,682,952..26,689,244	1457	1338	XM_022997346.1	
***OePIN2b***	NW_019245527	Unk	38678..41693	2646	1101	XM_023007658.1	
*PIN3* subfamily							
*OePIN3a*	NC_036237	1	14651491..14655627	2878	1896	XM_023021220.1	
*OePIN3b*	NC_036242	6	28,142,004..28,145,594	2924	1929	XM_023022745.1	
*OePIN3c*	NC_036244	8	17,824,834..17,828,738	3327	1929	XM_023026252.1	
*PIN5* subfamily							
*OePIN5a*	NC_036237	1	3,246,084..3,247,884	1623	1083	XM_022996510.1	
*OePIN5a*	NC_036237	1	3,254,921..3,256,721	1623	1083	XM_022996594.1	
*OePIN5b*	NC_036248	12	6,751,323..6,753,009	1572	1095	XM_023036296.1	
*OePIN5c*	NC_036255	19	6,145,679..6,147,551	1681	1062	X1: XM_022989965.1	-
					1041	X2: XM_022989967.1	AS_Int1
*OePIN5c*	NC_036255	19	6,156,663..6,158,539	1681	1062	X1: XM_022989968.1	-
					1041	X2: XM_022989969.1	AS_Int1
***OePIN5d***	NW_019241282	Unk	233..1706	1449	825	XM_023005297.1	
*PIN6* subfamily							
***OePIN6***	NC_036250	14	1,655,349..1,660,725	5323	1539	XM_023039423.1	
*PIN8* subfamily							
*OePIN8*	NC_036246	10	1,724,672..1,727,598	2377	1071	XM_023028238.1	

* This sequence lacks the exon 1 sequence that leads to the lack of the “Mem_trans” domain at the protein N-termini; for that reason was considered as a pseudogene. Genes in bold could be genes encoding non-functional proteins and for that reason were not included in the total number of functional genes. On these sequences, the hydrophobic segment that must appear conserved at both N and C-termini across PIN members, composed of several transmembrane helices, is incomplete or absent at least one termini (see results of that analysis in Supplementary Materials Figure S1); Event: corresponds to the event which gave rise to transcript variation; APA: Alternative PolyAdenylation; AS: Alternative Splicing. Transcript variant X1 corresponds to the transcript encoding complete sequence.

## Data Availability

The sequences of *Olea* *europaea* subsp. *europaea* var. *europaea* (cv. ‘Farga’) were retrieved from the olive genome database (http://denovo.cnag.cat/olive (accessed on 30 November 2017), Oe6 browser) and from *Olea* *europaea* subsp. *europaea* var. *sylvestris* from https://www.ncbi.nlm.nih.gov/genome/?term=Olea+europaea+var.+sylvestris+genome (accessed on 10 October 2018). RNA sequence data were achieved in European Nucleotide Archive databases (https://www.ebi.ac.uk/ena (accessed on 20 October 2019)) identified with the accession number PRJNA256033 (https://www.ebi.ac.uk/ena/data/view/PRJNA256033&portal=read_experiment (accessed on 20 October 2019)).

## Supplementary Materials

The following supporting information can be downloaded at: https://www.mdpi.com/article/10.3390/biology11071040/s1, Figure S1: Transmembrane topology of *O. europaea* subsp. *europaea* var. *sylvestris* PIN (OePIN) proteins are predicted by using the THMM automatic server (freely available at http://www.cbs.dtu.dk/services/TMHMM-2.0/ (accessed on 30 July 2019)). The predicted transmembrane helices are shown as red peaks; Figure S2: Transmembrane topology of *O. europaea* subsp. *europaea* var. *europaea* PIN (OePIN) proteins predicted by using the THMM automatic server (freely available at http://www.cbs.dtu.dk/services/TMHMM-2.0/ (accessed on 30 July 2019)). The predicted transmembrane helices are shown as red peaks; Figure S3: Pie-charts of GO terms for *OePINs* in the Biological Process (a), Cellular component (b), and Molecular Function (c) classes; Figure S4: Pie-charts of GO Biological Processes showing the distribution of upregulated and down-regulated genes at 2 and 7 days post-wounding (dpw); Figure S5: Pie-charts of GO Biological Processes showing the distribution of upregulated and down-regulated genes at 2 and 7 days post-infection (dpi) with *Verticillium dahliae*; Figure S6: Pie-charts of GO Biological Processes showing the distribution of upregulated and down-regulated genes at 1 and 10 days after exposure to cold stress (dac); Figure S7: Comparison of activated KEGG pathways in the modules identified through WGCNA analysis related to the three stress conditions evaluated (cold: blue module, wounding: turquoise module, *V. dahliae* infection: blue module); Table S1: Identification of PIN sequences used for phylogenetic studies. Sequences were retrieved from Phytozome (https://phytozome.jgi.doe.gov/pz/portal.html (accessed on 7 December 2018)); Table S2: Description of samples used and respective accession numbers (https://www.ebi.ac.uk/ena/data/view/PRJNA256033&portal=read_experiment (accessed on 20 October 2019)); Table S3: Information regarding size and exon/intron composition (number and sequence length) of *OePIN* genes in *Olea europaea* subsp. *europaea* var. *europaea* and var. *sylvestris*; Table S4: Information regarding PIN members of *Olea europaea* subsp. *euroapea* var. *europaea* (sequences retrieved from the whole genome databases available for cv. ’Farga’ at http://denovo.cnag.cat/genomes/olive/ (accessed on 30 November 2017)); Table S5: Pairwise differential expression results achieved in the different analysis (all comparisons were made considering the control sample, in case of Cold treatment and wounding the samples collected at T0, and in case of infection with *V. dahliae* the samples collected at the same timepoints from wounded plants).

## References

[1] Brackmann K., Qi J., Gebert M., Jouannet V., Schlamp T., Grünwald K., Wallner E.-S., Novikova D.D., Levitsky V.G., Agustí J. (2018). Spatial Specificity of Auxin Responses Coordinates Wood Formation. Nat. Commun..

[2] Rodriguez-Villalon A. (2016). Wiring a Plant: Genetic Networks for Phloem Formation in *Arabidopsis thaliana* Roots. New Phytol..

[3] An J., LIU X., LI H., YOU C., SHU J., WANG X., HAO Y. (2017). Molecular Cloning and Functional Characterization of MdPIN1 in Apple. J. Integr. Agric..

[4] Gou J., Strauss S.H., Tsai C.J., Fang K., Chen Y., Jiang X., Busov V.B. (2010). Gibberellins Regulate Lateral Root Formation in Populus through Interactions with Auxin and Other Hormones. Plant Cell.

[5] Palovaara J., Hallberg H., Stasolla C., Luit B., Hakman I. (2010). Expression of a Gymnosperm PIN Homologous Gene Correlates with Auxin Immunolocalization Pattern at Cotyledon Formation and in Demarcation of the Procambium during Picea Abies Somatic Embryo Development and in Seedling Tissues. Tree Physiol..

[6] Pahari S., Cormark R.D., Blackshaw M.T., Liu C., Erickson J.L., Schultz E.A. (2014). *Arabidopsis* UNHINGED Encodes a VPS51 Homolog and Reveals a Role for the GARP Complex in Leaf Shape and Vein Patterning. Development.

[7] Hakman I., Hallberg H., Palovaara J. (2009). The Polar Auxin Transport Inhibitor NPA Impairs Embryo Morphology and Increases the Expression of an Auxin Efflux Facilitator Protein PIN during Picea Abies Somatic Embryo Development. Tree Physiol..

[8] Petrásek J., Friml J. (2009). Auxin Transport Routes in Plant Development. Dev. Camb. Engl..

[9] Okada K., Ueda J., Komaki M.K., Bell C.J., Shimura Y. (1991). Requirement of the Auxin Polar Transport System in Early Stages of *Arabidopsis* Floral Bud Formation. Plant Cell.

[10] Salazar R., Pollmann S., Morales-Quintana L., Herrera R., Caparrós-Ruiz D., Ramos P. (2019). In Seedlings of *Pinus radiata*, Jasmonic Acid and Auxin Are Differentially Distributed on Opposite Sides of Tilted Stems Affecting Lignin Monomer Biosynthesis and Composition. Plant Physiol. Biochem..

[11] Campos M.D., Nogales A., Cardoso H.G., Kumar S.R., Nobre T., Sathishkumar R., Arnholdt-Schmitt B. (2016). Stress-Induced Accumulation of DcAOX1 and DcAOX2a Transcripts Coincides with Critical Time Point for Structural Biomass Prediction in Carrot Primary Cultures (*Daucus carota* L.). Front. Genet..

[12] Velada I., Grzebelus D., Lousa D., Soares C.M., Santos Macedo E., Peixe A., Arnholdt-Schmitt B., Cardoso H.G. (2018). AOX1-Subfamily Gene Members in *Olea europaea* Cv. “Galega Vulgar”—Gene Characterization and Expression of Transcripts during IBA-Induced in Vitro Adventitious Rooting. Int. J. Mol. Sci..

[13] Cardoso H.G., Campos M.C., Pais M.S., Peixe A. (2010). Use of Morphometric Parameters for Tracking Ovule and Microspore Evolution in Grapevine (*Vitis vinifera* L., Cv. “Aragonez”) and Evaluation of Their Potential to Improve in Vitro Somatic Embryogenesis Efficiency from Gametophyte Tissues. In Vitro Cell. Dev. Biol. Plant.

[14] Pires R., Cardoso H., Ribeiro A., Peixe A., Cordeiro A. (2020). Somatic Embryogenesis from Mature Embryos of *Olea europaea* L. Cv. ‘Galega Vulgar’ and Long-Term Management of Calli Morphogenic Capacity. Plants.

[15] Cardoso H.G., Arnholdt-Schmitt B., Lübberstedt T., Varshney R.K. (2013). Functional Marker Development Across Species in Selected Traits. Diagnostics in Plant Breeding.

[16] Arnholdt-Schmitt B., Ragonezi C., Cardoso H. (2016). Do Mitochondria Play a Central Role in Stress-Induced Somatic Embryogenesis?. Methods Mol. Biol. Clifton NJ.

[17] Korver R.A., Koevoets I.T., Testerink C. (2018). Out of Shape During Stress: A Key Role for Auxin. Trends Plant Sci..

[18] Blakeslee J.J., Spatola Rossi T., Kriechbaumer V. (2019). Auxin Biosynthesis: Spatial Regulation and Adaptation to Stress. J. Exp. Bot..

[19] Casanova-Sáez R., Mateo-Bonmatí E., Ljung K. (2022). Auxin Metabolism in Plants. Auxin Signaling: From Synthesis to Systems Biology.

[20] Finet C., Jaillais Y. (2012). AUXOLOGY: When Auxin Meets Plant Evo-Devo. Dev. Biol..

[21] Balzan S., Johal G.S., Carraro N. (2014). The Role of Auxin Transporters in Monocots Development. Front. Plant Sci..

[22] Grones P., Friml J. (2015). Auxin Transporters and Binding Proteins at a Glance. J. Cell Sci..

[23] Zhou J.-J., Luo J. (2018). The PIN-FORMED Auxin Efflux Carriers in Plants. Int. J. Mol. Sci..

[24] Hille S., Akhmanova M., Glanc M., Johnson A., Friml J. (2018). Relative Contribution of PIN-Containing Secretory Vesicles and Plasma Membrane PINs to the Directed Auxin Transport: Theoretical Estimation. Int. J. Mol. Sci..

[25] Friml J., Benková E., Blilou I., Wisniewska J., Hamann T., Ljung K., Woody S., Sandberg G., Scheres B., Jürgens G. (2002). AtPIN4 Mediates Sink-Driven Auxin Gradients and Root Patterning in *Arabidopsis*. Cell.

[26] Friml J., Vieten A., Sauer M., Weijers D., Schwarz H., Hamann T., Offringa R., Jürgens G. (2003). Efflux-Dependent Auxin Gradients Establish the Apical-Basal Axis of *Arabidopsis*. Nature.

[27] Mravec J., Skůpa P., Bailly A., Hoyerová K., Krecek P., Bielach A., Petrásek J., Zhang J., Gaykova V., Stierhof Y.-D. (2009). Subcellular Homeostasis of Phytohormone Auxin Is Mediated by the ER-Localized PIN5 Transporter. Nature.

[28] Paponov I.A., Teale W.D., Trebar M., Blilou I., Palme K. (2005). The PIN Auxin Efflux Facilitators: Evolutionary and Functional Perspectives. Trends Plant Sci..

[29] Wang Y., Chai C., Valliyodan B., Maupin C., Annen B., Nguyen H.T. (2015). Genome-Wide Analysis and Expression Profiling of the PIN Auxin Transporter Gene Family in Soybean (*Glycine max*). BMC Genom..

[30] Forestan C., Farinati S., Varotto S. (2012). The Maize PIN Gene Family of Auxin Transporters. Front. Plant Sci..

[31] Roumeliotis E., Kloosterman B., Oortwijn M., Visser R., Bachem C. (2013). The PIN Family of Proteins in Potato and Their Putative Role in Tuberization. Front. Plant Sci..

[32] Zhang Y., He P., Yang Z., Huang G., Wang L., Pang C., Xiao H., Zhao P., Yu J., Xiao G. (2017). A Genome-Scale Analysis of the PIN Gene Family Reveals Its Functions in Cotton Fiber Development. Front. Plant Sci..

[33] Hou M., Luo F., Wu D., Zhang X., Lou M., Shen D., Yan M., Mao C., Fan X., Xu G. (2021). OsPIN9, an Auxin Efflux Carrier, Is Required for the Regulation of Rice Tiller Bud Outgrowth by Ammonium. New Phytol..

[34] Huang X., Bai X., Guo T., Xie Z., Laimer M., Du D., Gbokie T., Zhang Z., He C., Lu Y. (2020). Genome-Wide Analysis of the PIN Auxin Efflux Carrier Gene Family in Coffee. Plants.

[35] Qi L., Chen L., Wang C., Zhang S., Yang Y., Liu J., Li D., Song J., Wang R. (2020). Characterization of the Auxin Efflux Transporter PIN Proteins in Pear. Plants.

[36] Adamowski M., Friml J. (2015). PIN-Dependent Auxin Transport: Action, Regulation, and Evolution. Plant Cell.

[37] Abdollahi Sisi N., Růžička K. (2020). ER-Localized PIN Carriers: Regulators of Intracellular Auxin Homeostasis. Plants.

[38] Kühn N., Serrano A., Abello C., Arce A., Espinoza C., Gouthu S., Deluc L., Arce-Johnson P. (2016). Regulation of Polar Auxin Transport in Grapevine Fruitlets (*Vitis vinifera* L.) and the Proposed Role of Auxin Homeostasis during Fruit Abscission. BMC Plant Biol..

[39] Song C., Zhang D., Zhang J., Zheng L., Zhao C., Ma J., An N., Han M. (2016). Expression Analysis of Key Auxin Synthesis, Transport, and Metabolism Genes in Different Young Dwarfing Apple Trees. Acta Physiol. Plant..

[40] Carraro N., Tisdale-Orr T.E., Clouse R.M., Knöller A.S., Spicer R. (2012). Diversification and Expression of the PIN, AUX/LAX, and ABCB Families of Putative Auxin Transporters in Populus. Front. Plant Sci..

[41] Grunewald W., Cannoot B., Friml J., Gheysen G. (2009). Parasitic Nematodes Modulate PIN-Mediated Auxin Transport to Facilitate Infection. PLOS Pathog..

[42] Pasternak T., Rudas V., Potters G., Jansen M.A.K. (2005). Morphogenic Effects of Abiotic Stress: Reorientation of Growth in *Arabidopsis thaliana* Seedlings. Environ. Exp. Bot..

[43] Shibasaki K., Uemura M., Tsurumi S., Rahman A. (2009). Auxin Response in *Arabidopsis* under Cold Stress: Underlying Molecular Mechanisms. Plant Cell.

[44] Ruedell C.M., de Almeida M.R., Fett-Neto A.G. (2015). Concerted Transcription of Auxin and Carbohydrate Homeostasis-Related Genes Underlies Improved Adventitious Rooting of Microcuttings Derived from Far-Red Treated *Eucalyptus globulus* Labill Mother Plants. Plant Physiol. Biochem..

[45] Velada I., Cardoso H., Porfirio S., Peixe A. (2020). Expression Profile of PIN-Formed Auxin Efflux Carrier Genes during IBA-Induced In Vitro Adventitious Rooting in *Olea europaea* L. Plants.

[46] Kumar S., Stecher G., Tamura K. (2016). MEGA7: Molecular Evolutionary Genetics Analysis Version 7.0 for Bigger Datasets. Mol. Biol. Evol..

[47] Tamura K., Nei M., Kumar S. (2004). Prospects for Inferring Very Large Phylogenies by Using the Neighbor-Joining Method. Proc. Natl. Acad. Sci. USA.

[48] Rambaut A. (2010). FigTree v1.3.1.

[49] Kohany O., Gentles A.J., Hankus L., Jurka J. (2006). Annotation, Submission and Screening of Repetitive Elements in Repbase: RepbaseSubmitter and Censor. BMC Bioinform..

[50] Hall T.A. (1999). BioEdit: A User-Friendly Biological Sequence Alignment Editor and Analysis Program for Windows 95/98/NT. Nucleic Acids Symp. Ser..

[51] Barghini E., Natali L., Giordani T., Cossu R.M., Scalabrin S., Cattonaro F., Šimková H., Vrána J., Doležel J., Morgante M. (2015). LTR Retrotransposon Dynamics in the Evolution of the Olive (*Olea europaea*) Genome. DNA Res..

[52] Nakai K., Kanehisa M. (1991). Expert System for Predicting Protein Localization Sites in Gram-Negative Bacteria. Proteins.

[53] Bennett T., Brockington S.F., Rothfels C., Graham S.W., Stevenson D., Kutchan T., Rolf M., Thomas P., Wong G.K.-S., Leyser O. (2014). Paralogous Radiations of PIN Proteins with Multiple Origins of Noncanonical PIN Structure. Mol. Biol. Evol..

[54] Leyva-Pérez M.d.l.O., Valverde-Corredor A., Valderrama R., Jiménez-Ruiz J., Muñoz-Merida A., Trelles O., Barroso J.B., Mercado-Blanco J., Luque F. (2015). Early and Delayed Long-Term Transcriptional Changes and Short-Term Transient Responses during Cold Acclimation in Olive Leaves. DNA Res..

[55] Jiménez-Ruiz J., Leyva-Pérez M.d.l.O., Schilirò E., Barroso J.B., Bombarely A., Mueller L., Mercado-Blanco J., Luque F. (2017). Transcriptomic Analysis of *Olea europaea* L. Roots during the *Verticillium dahliae* Early Infection Process. Plant Genome.

[56] Bolger A.M., Lohse M., Usadel B. (2014). Trimmomatic: A Flexible Trimmer for Illumina Sequence Data. Bioinformatics.

[57] Cruz F., Julca I., Gómez-Garrido J., Loska D., Marcet-Houben M., Cano E., Galán B., Frias L., Ribeca P., Derdak S. (2016). Genome Sequence of the Olive Tree, *Olea europaea*. GigaScience.

[58] Götz S., García-Gómez J.M., Terol J., Williams T.D., Nagaraj S.H., Nueda M.J., Robles M., Talón M., Dopazo J., Conesa A. (2008). High-Throughput Functional Annotation and Data Mining with the Blast2GO Suite. Nucleic Acids Res..

[59] Kanehisa M., Sato Y., Kawashima M., Furumichi M., Tanabe M. (2016). KEGG as a Reference Resource for Gene and Protein Annotation. Nucleic Acids Res..

[60] Langfelder P., Horvath S. (2008). WGCNA: An R Package for Weighted Correlation Network Analysis. BMC Bioinform..

[61] Barak L.S., Ménard L., Ferguson S.S., Colapietro A.M., Caron M.G. (1995). The Conserved Seven-Transmembrane Sequence NP(X)2,3Y of the G-Protein-Coupled Receptor Superfamily Regulates Multiple Properties of the Beta 2-Adrenergic Receptor. Biochemistry.

[62] Retzer K., Lacek J., Skokan R., Del Genio C.I., Vosolsobě S., Laňková M., Malínská K., Konstantinova N., Zažímalová E., Napier R.M. (2017). Evolutionary Conserved Cysteines Function as Cis-Acting Regulators of *Arabidopsis* PIN-FORMED 2 Distribution. Int. J. Mol. Sci..

[63] Julca I., Marcet-Houben M., Vargas P., Gabaldón T. (2018). Phylogenomics of the Olive Tree (*Olea europaea*) Reveals the Relative Contribution of Ancient Allo- and Autopolyploidization Events. BMC Biol..

[64] Mandáková T., Li Z., Barker M.S., Lysak M.A. (2017). Diverse Genome Organization Following 13 Independent Mesopolyploid Events in *Brassicaceae* Contrasts with Convergent Patterns of Gene Retention. Plant J..

[65] Kong H., Landherr L.L., Frohlich M.W., Leebens-Mack J., Ma H., DePamphilis C.W. (2007). Patterns of Gene Duplication in the Plant SKP1 Gene Family in Angiosperms: Evidence for Multiple Mechanisms of Rapid Gene Birth. Plant J..

[66] Rao G., Zhang J., Liu X., Lin C., Xin H., Xue L., Wang C. (2021). De Novo Assembly of a New *Olea europaea* Genome Accession Using Nanopore Sequencing. Hortic. Res..

[67] Yang C., Wang D., Zhang C., Kong N., Ma H., Chen Q. (2019). Comparative Analysis of the PIN Auxin Transporter Gene Family in Different Plant Species: A Focus on Structural and Expression Profiling of PINs in *Solanum tuberosum*. Int. J. Mol. Sci..

[68] Velada I., Cardoso H.G., Ragonezi C., Nogales A., Ferreira A., Valadas V., Arnholdt-Schmitt B. (2016). Alternative Oxidase Gene Family in *Hypericum perforatum* L.: Characterization and Expression at the Post-Germinative Phase. Front. Plant Sci..

[69] Cardoso H.G., Nogales A., Frederico A.M., Svensson J.T., Macedo E.S., Valadas V., Arnholdt-Schmitt B. (2015). Exploring AOX Gene Diversity. Alternative Respiratory Pathways in Higher Plants.

[70] Knowles D.G., McLysaght A. (2006). High Rate of Recent Intron Gain and Loss in Simultaneously Duplicated *Arabidopsis* Genes. Mol. Biol. Evol..

[71] Fiume E., Christou P., Gianì S., Breviario D. (2004). Introns Are Key Regulatory Elements of Rice Tubulin Expression. Planta.

[72] Zhang J., Gu H., Dai H., Zhang Z., Miao M. (2020). Alternative Polyadenylation of the Stacyose Synthase Gene Mediates Source-Sink Regulation in Cucumber. J. Plant Physiol..

[73] Tian B., Manley J.L. (2017). Alternative Polyadenylation of MRNA Precursors. Nat. Rev. Mol. Cell Biol..

[74] Tu Z., Shen Y., Wen S., Liu H., Wei L., Li H. (2021). A Tissue-Specific Landscape of Alternative Polyadenylation, LncRNAs, TFs, and Gene Co-Expression Networks in *Liriodendron chinense*. Front. Plant Sci..

[75] Choi J.Y., Lee Y.C.G. (2020). Double-Edged Sword: The Evolutionary Consequences of the Epigenetic Silencing of Transposable Elements. PLoS Genet..

[76] Negi P., Rai A.N., Suprasanna P. (2016). Moving through the Stressed Genome: Emerging Regulatory Roles for Transposons in Plant Stress Response. Front. Plant Sci..

[77] Thieme M., Bucher E., Mirouze M., Bucher E., Gallusci P. (2018). Chapter Six—Transposable Elements as Tool for Crop Improvement. Advances in Botanical Research.

[78] Giles N.M., Watts A.B., Giles G.I., Fry F.H., Littlechild J.A., Jacob C. (2003). Metal and Redox Modulation of Cysteine Protein Function. Chem. Biol..

[79] Xia X.-J., Zhou Y.-H., Shi K., Zhou J., Foyer C.H., Yu J.-Q. (2015). Interplay between Reactive Oxygen Species and Hormones in the Control of Plant Development and Stress Tolerance. J. Exp. Bot..

[80] Cardoso H., Peixe A., Bellini C., Porfírio S., Druege U. (2022). Editorial: Advances on the Biological Mechanisms Involved in Adventitious Root Formation: From Signaling to Morphogenesis. Front. Plant Sci..

[81] Wilkins K.A., Matthus E., Swarbreck S.M., Davies J.M. (2016). Calcium-Mediated Abiotic Stress Signaling in Roots. Front. Plant Sci..

[82] Aldon D., Mbengue M., Mazars C., Galaud J.-P. (2018). Calcium Signalling in Plant Biotic Interactions. Int. J. Mol. Sci..

[83] Tognetti V.B., Mühlenbock P., Van Breusegem F. (2012). Stress Homeostasis—The Redox and Auxin Perspective. Plant Cell Environ..

[84] Grunewald W., Friml J. (2010). The March of the PINs: Developmental Plasticity by Dynamic Polar Targeting in Plant Cells. EMBO J..

[85] Campos M.D., Campos C., Nogales A., Cardoso H. (2021). Carrot AOX2a Transcript Profile Responds to Growth and Chilling Exposure. Plants.

[86] Fradin E.F., Thomma B.P.H.J. (2006). Physiology and Molecular Aspects of *Verticillium* Wilt Diseases Caused by *V. dahliae* and *V. albo-atrum*. Mol. Plant Pathol..

[87] Prieto P., Navarro-Raya C., Valverde-Corredor A., Amyotte S.G., Dobinson K.F., Mercado-Blanco J. (2009). Colonization Process of Olive Tissues by *Verticillium dahliae* and Its in Planta Interaction with the Biocontrol Root Endophyte *Pseudomonas fluorescens* PICF7. Microb. Biotechnol..

[88] Meents A.K., Furch A.C.U., Almeida-Trapp M., Özyürek S., Scholz S.S., Kirbis A., Lenser T., Theißen G., Grabe V., Hansson B. (2019). Beneficial and Pathogenic Arabidopsis Root-Interacting Fungi Differently Affect Auxin Levels and Responsive Genes During Early Infection. Front. Microbiol..

[89] Hoffmann M., Hentrich M., Pollmann S. (2011). Auxin-Oxylipin Crosstalk: Relationship of AntagonistsF. J. Integr. Plant Biol..

