# Supplementary material for: Understanding the Role of PIN Auxin Carrier Genes under Biotic and Abiotic Stresses in Olea europaea L
Source: Biology (Basel). 2022 Jul 11;11(7):1040. doi: 10.3390/biology11071040 (PMC9312197; doi:10.3390/biology11071040)
Supplement: Supplementary file 1 [file biology-11-01040-s001.zip › Supplementary Table S1.pdf]

Supplementary Table S1. Identification of PIN sequences used for phylogenetic studies.

	Accession numbers	Proposed nomenclature	Previous nomenclature
***Arabidopsis thaliana (Arabidopsis thaliana* TAIR10 – *Phytozome* 12)**	 <br>AT1G73590.1<br>AT5G57090.1<br>AT1G70940.1<br>AT2G01420.2<br>AT5G16530.1<br>AT1G77110.1<br>AT1G23080.1<br>AT5G15100.1		*Bennett et al. (2014), Wang et al. (2015)*<br>ArathPIN1<br>ArathPIN2<br>ArathPIN3<br>ArathPIN4<br>ArathPIN5<br>ArathPIN6<br>ArathPIN7<br>ArathPIN8
Arabidopsis lyrata (Arabidopsis lyrata* v2.1 – *Phytozome 12)	 AL2G33340.t1<br>AL8G32990.t1<br>AL2G30460.t1<br>AL5G10390.t1<br>AL6G27480.t1<br>AL2G37280.t1<br>AL1G36650.t1<br>AL6G25920.t2		*Bennett et al. (2014)*<br>AralyPIN1<br>AralyPIN2<br>AralyPIN3<br>AralyPIN4<br>AralyPIN5<br>AralyPIN6<br>AralyPIN7<br>AralyPIN8
Capsella grandiflora (Capsella grandiflora v1.1 – Phytozome 12)	Cagra.17308s0005.1<br>Cagra.1569s0017.1<br>Cagra.0147s0051.1<br>Cagra.1451s0018.1<br>Cagra.0434s0020.1<br>Cagra.0605s0108.1<br>Cagra.1036s0047.1	CagraPIN1<br>CagraPIN2<br>CagraPIN3<br>CagraPIN4<br>CagraPIN5<br>CagraPIN7<br>CagraPIN8	
Capsella rubella (Capsella rubella v1.0 – Phytozome 12)	Carubv10019970m<br>Carubv10026053m<br>Carubv10019932m<br>Carubv10016856m<br>Carubv10001290m<br>Carubv10020056m<br>Carubv10008599m<br>Carubv10003844m	CarubPIN1<br>CarubPIN2<br>CarubPIN3<br>CarubPIN4<br>CarubPIN5<br>CarubPIN6<br>CarubPIN7<br>CarubPIN8	
Glycine max (Glycine max - Plaza v2.5)	 GM08G05900<br>GM07G11550<br>GM09G30700<br>GM03G28130<br>GM19G30900<br>GM13G00390<br>GM17G06460<br>GM07G34190<br>GM20G01760<br>GM07G22340<br>GM09G20580<br>GM09G38700<br>GM18G47630<br>GM13G09030<br>GM14G27900<br>GM05G23180<br>GM17G16870<br>GM18G49080<br>GM09G06970		*Wang et al. (2015)*<br>GmPIN1a<br>GmPIN1b<br>GmPIN1c<br>GmPIN1d<br>GmPIN1e<br>GmPIN2a<br>GmPIN2b<br>GmPIN3a<br>GmPIN3b<br>GmPIN3c<br>GmPIN3d<br>GmPIN5a<br>GmPIN5b<br>GmPIN6a<br>GmPIN6b<br>GmPIN8a<br>GmPIN8b<br>GmPIN8d<br>GmPIN9a

	GM09G13500<br>GM15G25690	GmPIN9b	GmPIN9c<br>GmPIN9d
***Manihot esculenta* (*Manihot esculenta v6.1 – Phytozome 12*)**	Manes.14G171100.1<br>Manes.06G006800.1<br>Manes.08G060200.1<br>Manes.06G000100.1<br>Manes.17G077100.1<br>Manes.15G127700.1<br>Manes.09G184600.1<br>Manes.08G104400.1<br>Manes.18G087300.1<br>Manes.S018500.1	ManesPIN1a<br>ManesPIN1b<br>ManesPIN1c<br>ManesPIN2<br>ManesPIN3a<br>ManesPIN3b<br>ManesPIN5a<br>ManesPIN5b<br>ManesPIN6<br>ManesPIN8	
***Mimulus guttatus* (*Mimulus guttatus v2.0 – Phytozome 12*)**	Migut.D01545.1<br>Migut.I00299.1<br>Migut.J01639.1<br>Migut.J01508.1<br>Migut.E00429.1<br>Migut.L00994.1<br>Migut.F02141.1<br>Migut.F02142.1<br>Migut.C01042.1<br>Migut.H00890.1	MimguPIN1a<br>MimguPIN1b<br>MimguPIN1c<br><br>MimguPIN3a<br>MimguPIN3b<br>MimguPIN5a<br>MimguPIN5b	*Bennett et al. (2014)*<br>MimguPIN1α<br>MimguPIN1β<br>MimguPIN11<br>MimguPIN2<br>MimguPIN3α<br>MimguPIN3β<br>MimguPIN5α<br>MimguPIN5β<br>MimguPIN6<br>MimguPIN8
***Solanum lycopersicon* (*Solanum lycopersicon iTAG2.4 (Tomato) - Phytozome 12*)**	Solyc03g118740.2.1<br>Solyc10g078370.1.1<br>Solyc10g080880.1.1<br>Solyc07g006900.1.1<br>Solyc05g008060.2.1<br>Solyc04g007690.2.1<br>Solyc01g068410.2.1<br>Solyc04g056620.1.1<br>Solyc06g059730.1.1<br>Solyc02g087660.2.1	SolycPIN1a<br>SolycPIN1b<br>SolycPIN1c<br><br>SolycPIN3a<br>SolycPIN3b<br>SolycPIN5a<br>SolycPIN5b	*Bennett et al. (2014)*<br>SolycPIN1<br>SolycPIN11α<br>SolycPIN11β<br>SolycPIN2<br>SolycPIN3α<br>SolycPIN3β<br>SolycPIN5α<br>SolycPIN5β<br>SolycPIN6<br>SolycPIN8
***Solanum tuberosum* (*Solanum tuberosum v4.03 – Phytozome 12*)**	PGSC0003DMT400014752<br>PGSC0003DMT400021600<br>PGSC0003DMT400072459<br>PGSC0003DMT400048251<br>PGSC0003DMT400078330<br>PGSC0003DMT400015267<br>PGSC0003DMT400046253<br>PGSC0003DMT400027309<br>PGSC0003DMT400079013<br>PGSC0003DMT400003569	SotubPIN1a<br>SotubPIN1b<br>SotubPIN1c<br>SotubPIN2<br>SotubPIN3a<br>SotubPIN3b<br>SotubPIN5a<br>SotubPIN5b<br>SotubPIN6<br>SotubPIN8	
***Medicago truncatula* (*Medicago truncatula Mt4.OU1 – Phytozome 12*)**	Medtr6g069510.1<br>Medtr8g107360.1<br>Medtr7g089360.1<br>Medtr4g127100.1<br>Medtr4g127090.1<br>Medtr4g084870.1<br>Medtr1g030890.1<br>Medtr7g079720.1<br>Medtr2g038470.1<br>Medtr1g029190.1<br>Medtr6g011400.1<br>Medtr7g009370.1		*Wang et al. (2015)*<br>MtPIN1a<br>MtPIN1b<br>MtPIN1c<br>MtPIN2a<br>MtPIN2b<br>MtPIN3a<br>MtPIN3b<br>MtPIN5a<br>MtPIN5b<br>MtPIN6<br>MtPIN8a<br>MtPIN8b

***Phaseolus vulgaris* (*Phaseolus vulgaris* v2.1 – Phytozome 12)**	Phvul.002G330300.1<br>Phvul.004G150600.1<br>Phvul.011G164500.1<br>Phvul.001G121100.1<br>Phvul.003G139600.1<br>Phvul.002G094000.1<br>Phvul.003G032500.1<br>Phvul.008G055200.1<br>Phvul.001G063900.1<br>Phvul.004G026500.1<br>Phvul.008G043700.1<br>Phvul.009G210900.1<br>Phvul.009G234000.1<br>Phvul.009G234100.1<br>Phvul.009G234200.1<br>Phvul.009G208200.1		*Wang et al. (2015)*<br>PvPIN1a<br>PvPIN1b<br>PvPIN1c<br>PvPIN1d<br>PvPIN2<br>PvPIN3a<br>PvPIN3b<br>PvPIN5<br>PvPIN6<br>PvPIN8a<br>PvPIN8b<br>PvPIN9a<br>PvPIN9b<br>PvPIN9c<br>PvPIN9d<br>PvPIN9e
