# Supplementary material for: Understanding the Role of PIN Auxin Carrier Genes under Biotic and Abiotic Stresses in Olea europaea L
Source: Biology (Basel). 2022 Jul 11;11(7):1040. doi: 10.3390/biology11071040 (PMC9312197; doi:10.3390/biology11071040)
Supplement: Supplementary file 1 [file biology-11-01040-s001.zip › Supplementary Table S2.pdf]

Supplementary Table S2. Description of samples used and respective accession numbers (https://www.ebi.ac.uk/ena/data/view/PRJNA256033&portal=read_experiment).

Sample	Biosample accession number	SRA accession number
Unwounded plant roots	SAMN02937426	SRR1525051<br>SRR1525052
Wounded roots_2dpw	SAMN02937429	SRR1524949<br>SRR1524950
Wounded roots_7dpw	SAMN02937430	SRR1524951<br>SRR1524952
Uninfected plant roots	SAMN02937426	SRR1525051<br>SRR1525052
Infected roots with V. dahliae_2dpi	SAMN02937431	SRR1525086<br>SRR1525087
Infected roots with V. dahliae_7dpi	SAMN02937432	SRR1525113<br>SRR1525114
Unstressed plant leaves	SAMN02937434	SRR1525224<br>SRR1525226
Cold-stressed leaves_1dac	SAMN02937435	SRR1525284<br>SRR1525285
Cold-stressed leaves_10dac	SAMN02937436	SRR1525286<br>SRR1525287

dpw: pays post wounding, dpi: days post inoculation, dac: days after cold exposure
