# Supplementary material for: Understanding the Role of PIN Auxin Carrier Genes under Biotic and Abiotic Stresses in Olea europaea L
Source: Biology (Basel). 2022 Jul 11;11(7):1040. doi: 10.3390/biology11071040 (PMC9312197; doi:10.3390/biology11071040)
Supplement: Supplementary file 1 [file biology-11-01040-s001.zip › Supplementary Table S3.pdf]

Gene	ID	ORF (bp)	Gene (bp)	No. exons	Exons lenght (bp)							Introns lenght (bp)					
					1	2	3	4	5	6	7	1	2	3	4	5	6
Olea europaea subsp. europaea var. euroapea																	
OePIN1a	OE6A110180P1	1794	3287	6	1168	-	241	86	158	77	67	79	-	130	**1115**	89	77
OePIN1b	OE6A100299P2	1794	3818	6	1168	-	241	86	158	77	67	88	-	81	**1104**	654	94
OePIN1c	OE6A008174P1	1776	3192	6	1144	-	247	86	158	77	67	87	-	92	309	826	99
OePIN1d	OE6A056306P2	1788	2853	6	1132	-	271	86	158	77	67	453	-	84	198	108	219
OePIN1e	OE6A046725P1	1788	2746	6	1132	-	271	86	158	77	67	445	-	87	193	102	128
OePIN1e	OE6A036288P1	1788	2740	6	1132	-	271	86	158	77	67	443	-	83	193	102	128
OePIN2a	OE6A080671P3	1872	3332	6	1231	-	256	86	158	77	67	197	-	487	319	291	163
OePIN2b	OE6A029229P1	1869	9225	6	1228	-	256	86	158	77	67	130	-	577	**6370**	165	111
OePIN3a	OE6A013411P1	1896	2873	6	1231	-	280	86	158	77	67	90	-	98	510	187	89
OePIN3b	OE6A121027P4	1929	2927	6	1255	-	289	86	158	77	67	131	-	89	456	218	101
OePIN3c	OE6A040519P1	1929	2897	6	1255	-	289	86	158	77	67	139	-	91	410	227	98
OePIN3c	OE6A094847P1	1929	2881	6	1255	-	289	86	158	77	67	140	-	91	403	228	87
OePIN5a	OE6A089248P2	1149	1689	5	758	-	-	86	158	77	73	96	-	-	226	112	103
OePIN5b	OE6A062743P1	1095	1567	5	704	-	-	86	158	77	73	79	-	-	176	110	104
OePIN5c	OE6A015595P2	1062	1681	5	671	-	-	86	158	77	73	121	-	-	102	312	81
OePIN5d	OE6A086522P1	1104	1736	5	713	-	-	86	158	77	73	140	-	-	112	286	91
OePIN5e	OE6A029376P1	1089	1713	5	698	-	-	86	158	77	73	140	-	-	103	286	92
OePIN6	OE6A074435P2	1584	5086	7	425	572	202	86	158	77	67	695	**2492**	74	78	72	88
OePIN8	OE6A113148P1	1071	2376	5	508	178	-	-	244	77	67	1080	66	-	-	69	87
Olea europaea subsp. europaea var. sylvestris																	
OePIN1a	XM_023034033.1	1794	3288	6	1168	-	241	86	158	77	67	79	-	131	1115	89	77
[1]*OePIN1b*	XM_023030314.1	1530	1697	3	1168	-	241	124	-	-	-	83	-	81	-	-	-
OePIN1c	XM_022985718.1	1776	3187	6	1144	-	247	86	158	77	67	87	-	92	297	833	99
OePIN1d	XM_023032854.1	1800	3639	6	1156	-	259	86	158	77	67	462	-	84	198	108	984
OePIN1e	XM_022996599.1	1788	2741	6	1132	-	271	86	158	77	67	445	-	83	192	102	128
[2]*OePIN2a*	XM_022997346.1	1338	1457	2	1228	-	113	-	-	-	-	116	-	-	-	-	-
[3]*OePIN2b*	XM_023007658.1	1101	2646	7	417	123	110	144	166	77	67	289	92	292	435	296	138
OePIN3a	XM_023021220.1	1896	2878	6	1231	-	280	86	158	77	67	90	-	98	515	187	89
OePIN3b	XM_023022745.1	1929	2924	6	1255	-	289	86	158	77	67	131	-	89	456	218	98
OePIN3c	XM_023026252.1	1929	3327	6	1255	-	289	86	158	77	67	140	-	91	849	228	87
OePIN5a	XM_022996510.1	1083	1623	5	692	-	-	86	158	77	73	140	-	-	103	286	92
OePIN5a	XM_022996594.1	1083	1623	5	692	-	-	86	158	77	73	96	-	-	226	112	103
OePIN5b	XM_023036296.1	1095	1572	5	704	-	-	86	158	77	73	79	-	-	176	110	109
OePIN5c	XM_022989965.1	1062	1681	5	671	-	-	86	158	77	73	121	-	-	102	312	81
OePIN5c	XM_022989968.1	1062	1681	5	671	-	-	86	158	77	73	121	-	-	102	312	81
OePIN5d	XM_023005297.1	825	1449	5	434	-	-	86	158	77	73	140	-	-	103	286	92
OePIN6	XM_023039423.1	1539	5323	6	425	572	202	86	142	-	115	695	2492	74	78	-	442
OePIN8	XM_023028238.1	1071	2377	5	508	178	-	-	244	77	67	1081	66	-	-	69	87

Supplementary Table S3. Information regarding size and exon/intron composition (number and sequence length) of *OePIN* genes in *Olea europaea* subsp. *europaea* var. *europaea* and var. *sylvestris*.

[1]Sequence interrupted due to an APA located at intron 3 which leads to a truncated protein sequence.
[2]*OePIN2a* – scaffold NC_036238.1 with 4541 Ns within the sequence that leads to the absence of the “Mem_tans” domain at the protein C-termini. Functionality of protein encoded by this gene sequence must to be confirmed.
