# Supplementary material for: Understanding the Role of PIN Auxin Carrier Genes under Biotic and Abiotic Stresses in Olea europaea L
Source: Biology (Basel). 2022 Jul 11;11(7):1040. doi: 10.3390/biology11071040 (PMC9312197; doi:10.3390/biology11071040)
Supplement: Supplementary file 1 [file biology-11-01040-s001.zip › Supplementary Table S4.pdf]

Supplementary Table S4. Information regarding PIN members of *Olea europaea* subsp. *euroapea* var. *europaea* (sequences retrieved from the whole genome databases available for cv.'Farga' at http://denovo.cnag.cat/genomes/olive/).

Gene	Locus ID	Protein				Number of transmembrane helices	Hydrophilic loop size (bp)	SP length (location: affirmative rate)
		Length (aa)	MW (kDa)	PI	Hydrophobic amino acids			
OePIN1a	OE6A110180P1	598	65.573	8.82	223	9 (5/4)	330	26 (PM: 0.640)
OePIN1b	OE6A100299P2	598	65.797	9.15	225	9 (5/4)	330	26 (PM: 0.640)
OePIN1c	OE6A008174P1	592	65.290	8.70	225	9 (5/4)	330	26 (PM: 0.640)
OePIN1d	OE6A056306P2	596	65.088	8.98	225	8 (4/4)	330	26 (PM: 0.640)
OePIN1e	OE6A046725P1	596	64.710	9.15	223	8 (4/4)	330	26 (PM: 0.640)
OePIN1e	OE6A036288P1	596	64.742	9.15	223	8 (4/4)	330	26 (PM: 0.640)
OePIN2a	OE6A080671P3	624	68.045	9.05	246	10 (5/5)	323	26 (PM: 0.640)
OePIN2b	OE6A029229P1	623	67.954	8.03	247	10 (5/5)	323	26 (ER: 0.685)
OePIN3a	OE6A013411P1	632	69.004	7.56	243	9 (5/4)	362	26 (PM: 0.640)
OePIN3b	OE6A121027P4	643	70.271	8.00	250	9 (5/4)	362	26 (PM: 0.640)
OePIN3c	OE6A040519P1	643	70.348	8.06	249	9 (5/4)	362	26 (PM: 0.640)
OePIN3c	OE6A094847P1	643	70.319	8.12	249	9 (5/4)	362	26 (PM: 0.640)
OePIN5a	OE6A089248P2	361	40.224	6.90	167	8 (4/4)	85	46 (PM: 0.600)
OePIN5b	OE6A062743P1	365	40.824	6.91	170	8 (4/4)	66	24 (ER: 0.685)
OePIN5c	OE6A015595P2	354	38.796	8.30	161	8 (4/4)	46	26 (ER: 0.685)
OePIN5d	OE6A086522P1	368	40.323	6.03	170	9 (5/4)	61	24 (ER: 0.685)
OePIN5e	OE6A029376P1	363	39.788	6.49	167	8 (4/4)	55	24 (ER: 0.685)
OePIN6	OE6A074435P2	528	58.644	8.09	216	9 (5/4)	303	26 (ER: 0.685)
OePIN8	OE6A113148P1	357	38.910	9.61	178	8 (4/4)	52	61 (ER: 0.685)

Protein length, molecular weight and isoelectric point were determined by using the EditSeq from Lasergene 7software (DNASTAR, Madison, WI,ISA). Transmembrane helices and topology of OePIN proteins was predicted at the THMM automatic server, freely available at https://services.healthtech.dtu.dk/service.php?TMHMM-2.0. Signal peptide (SP) was predicted by PSORT (http://psort1.hgc.jp/form.html).
