# Supplementary material for: Understanding the Role of PIN Auxin Carrier Genes under Biotic and Abiotic Stresses in Olea europaea L
Source: Biology (Basel). 2022 Jul 11;11(7):1040. doi: 10.3390/biology11071040 (PMC9312197; doi:10.3390/biology11071040)
Supplement: Supplementary file 1 [file biology-11-01040-s001.zip › Supplementary Table S5.pdf]

Supplementary Table S5. Pairwise differential expression results achieved in the different analysis (all comparisons were made considering the control sample, in case of Cold treatment and wounding the samples collected at T0, and in case of infection with *V. dahliae* the samples collected at the same timepoints from wounded plants).

	**Wounding**		***V. dahliae***		**Cold**	
Timepoint (days)	2	7	2	7	1	10
N. of features after filtering	45532	45534	43051	43171	40213	44126
up-regulated	5698	2857	6754	6116	2240	4188
down-regulated	8207	3933	5482	7953	2716	4394
