# Supplementary figures and images for: Understanding the Role of PIN Auxin Carrier Genes under Biotic and Abiotic Stresses in Olea europaea L
Source: Biology (Basel). 2022 Jul 11;11(7):1040. doi: 10.3390/biology11071040 (PMC9312197; doi:10.3390/biology11071040)

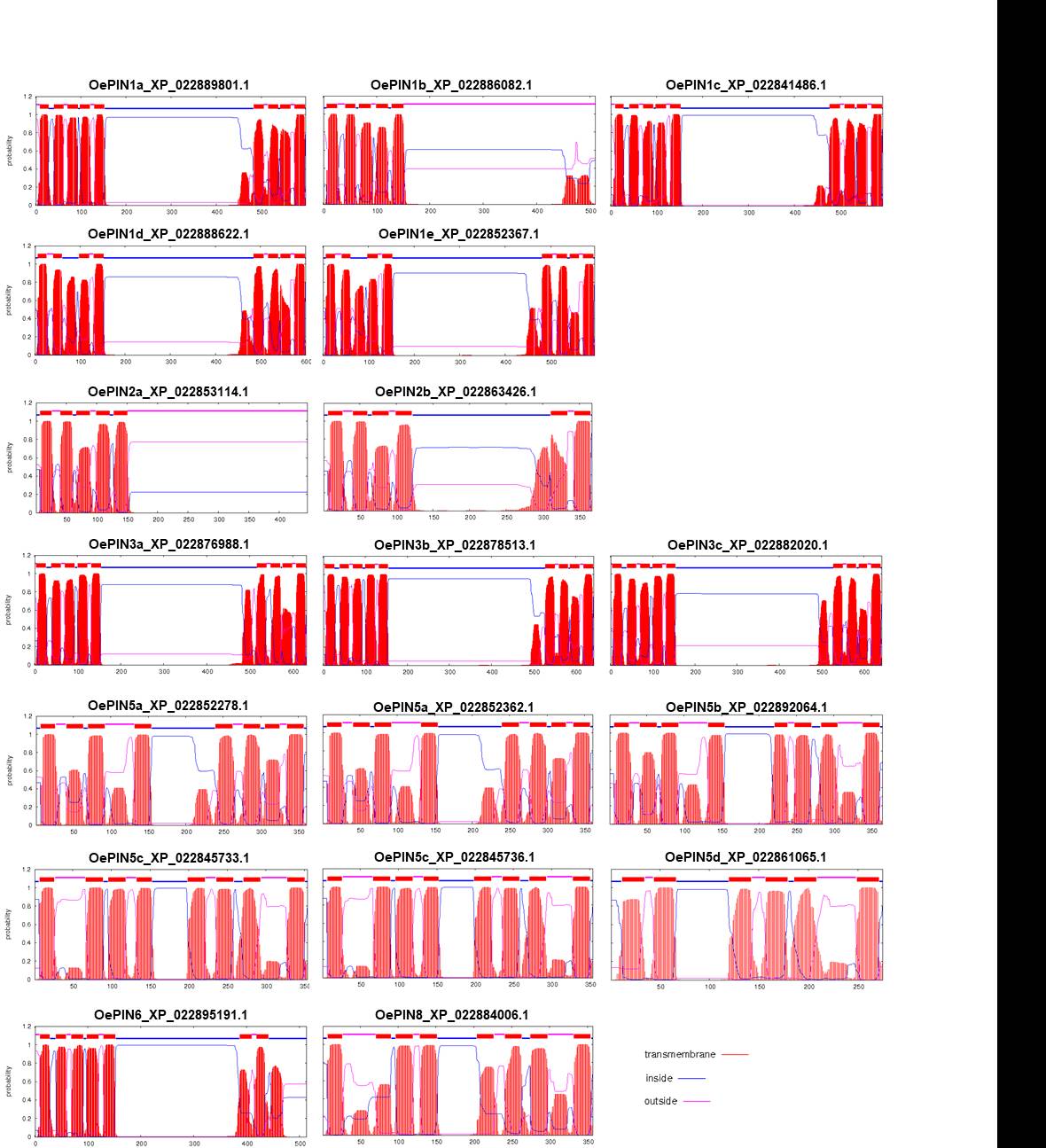

Supplement: Supplementary file 1 [file biology-11-01040-s001.zip › Supplementary FigS1.jpg]

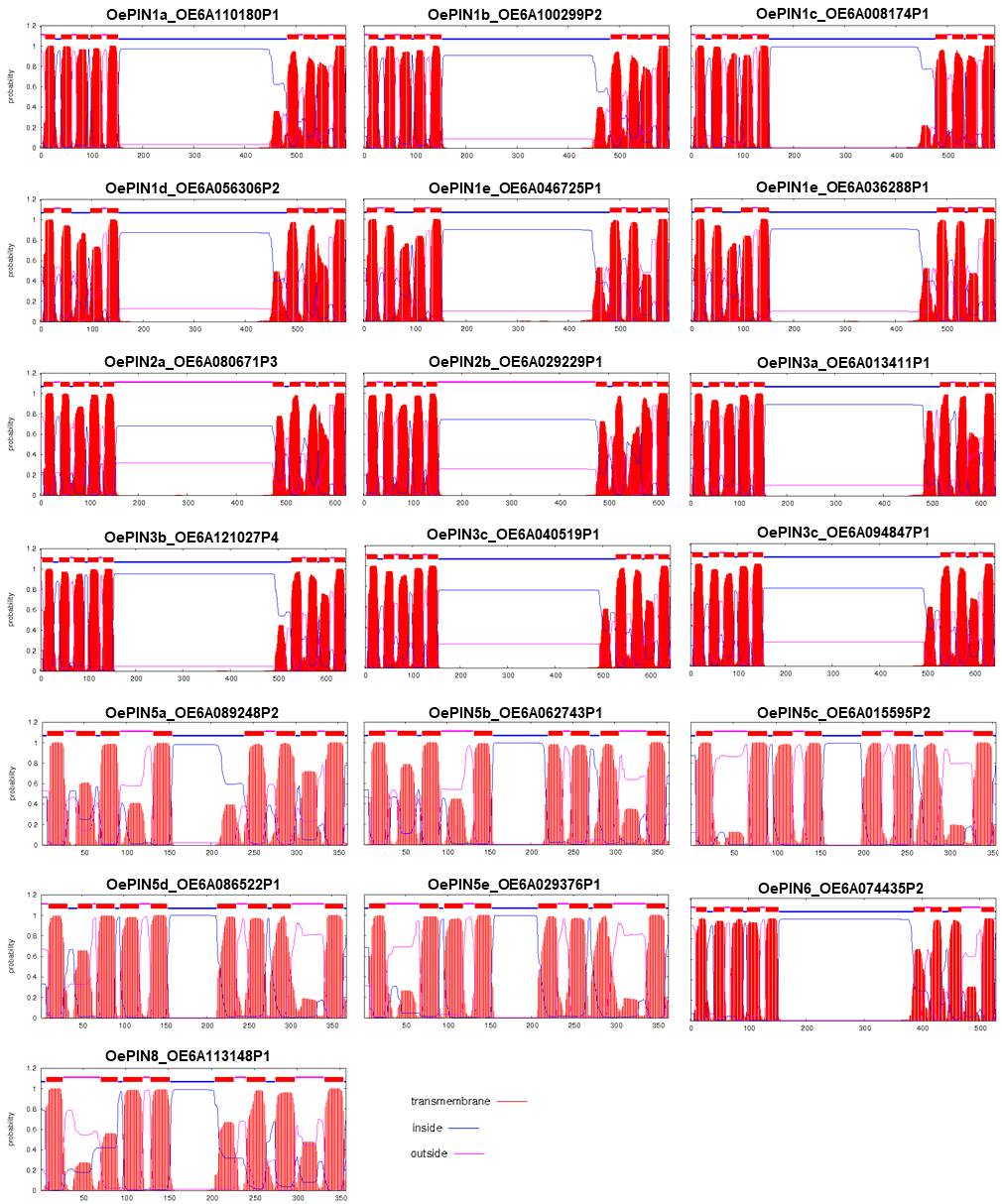

Supplement: Supplementary file 1 [file biology-11-01040-s001.zip › Supplementary FigS2.jpg]

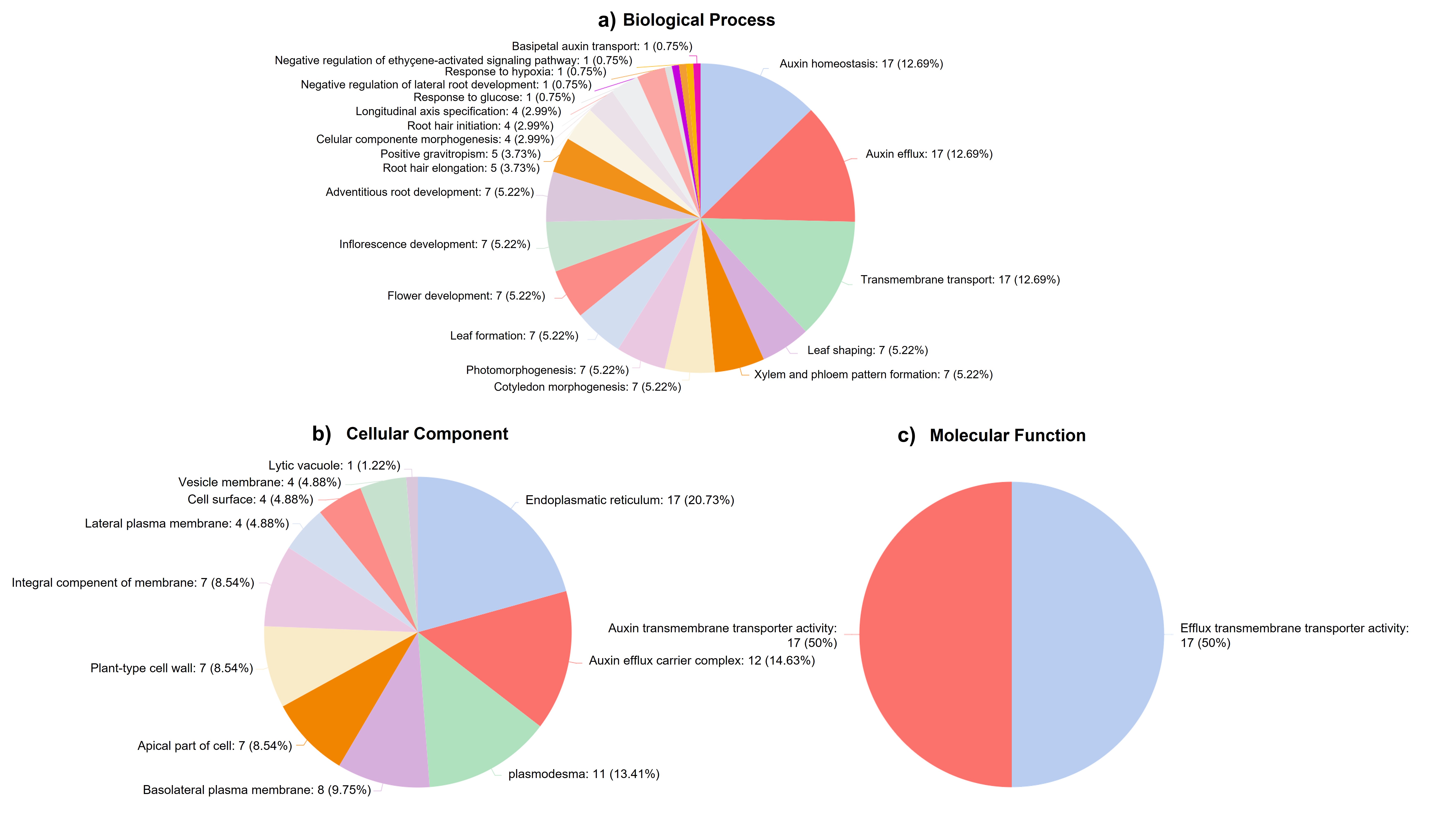

Supplement: Supplementary file 1 [file biology-11-01040-s001.zip › Supplementary FigS3.jpg]

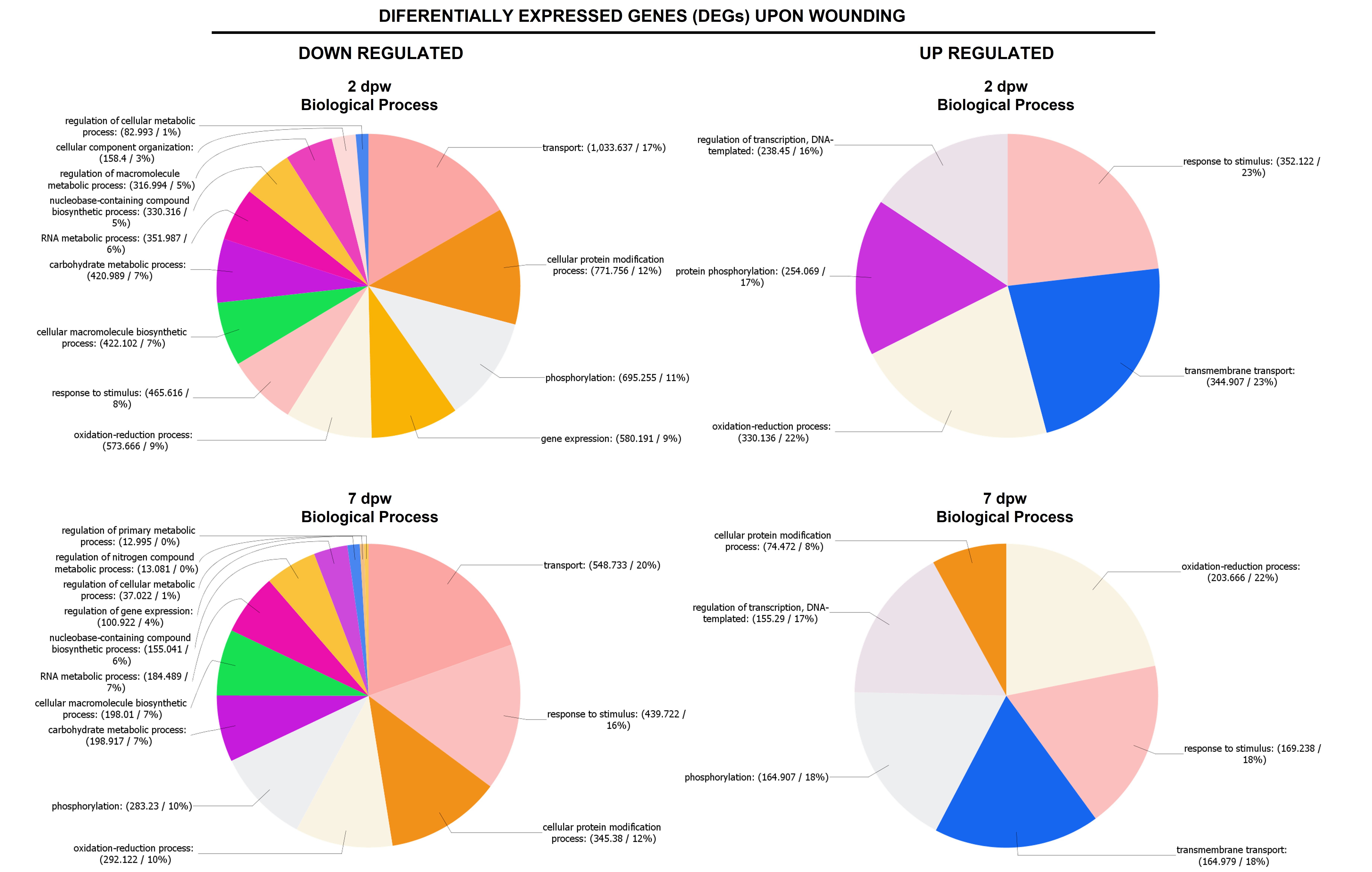

Supplement: Supplementary file 1 [file biology-11-01040-s001.zip › Supplementary FigS4.jpg]

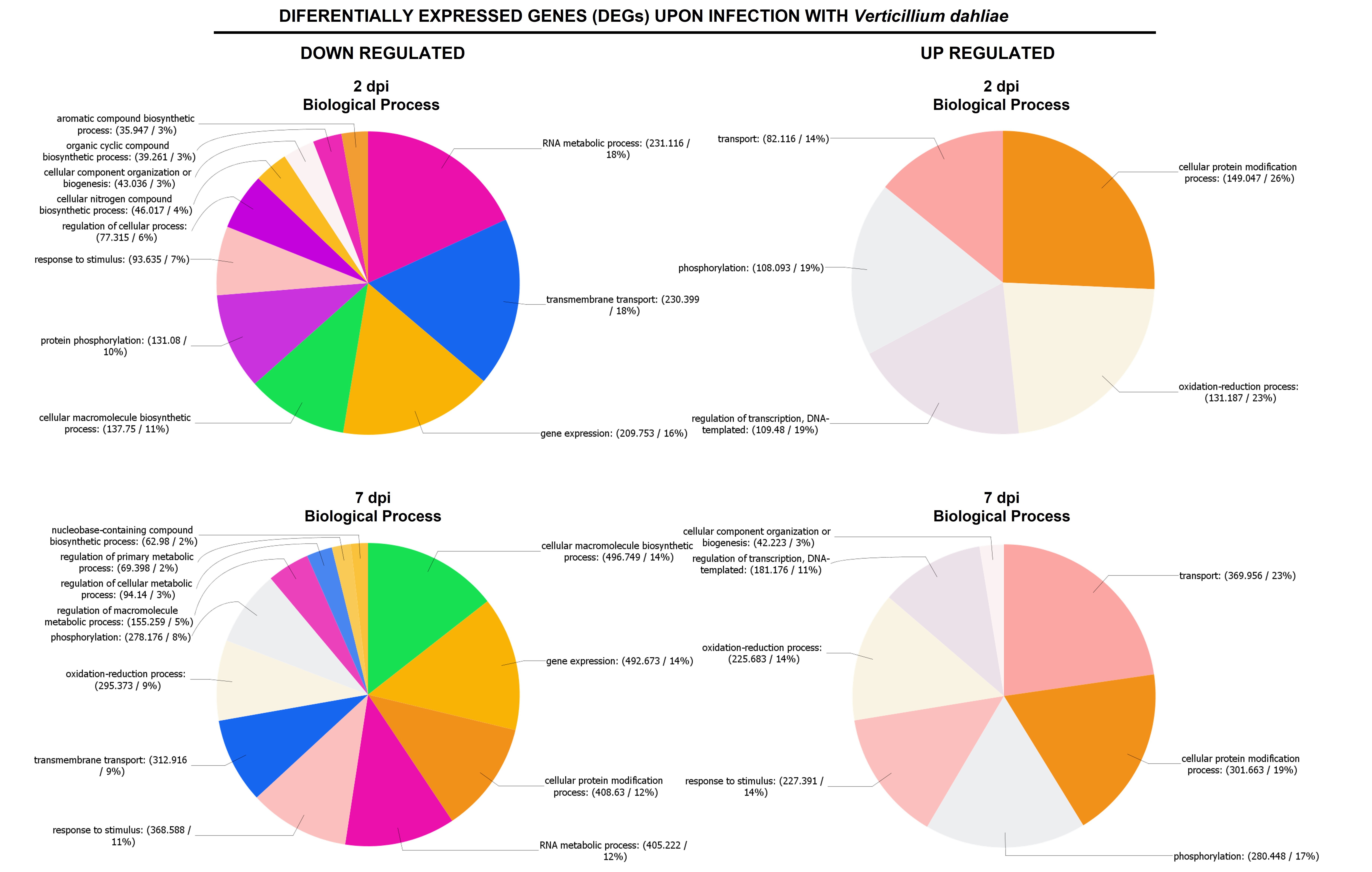

Supplement: Supplementary file 1 [file biology-11-01040-s001.zip › Supplementary FigS5.jpg]

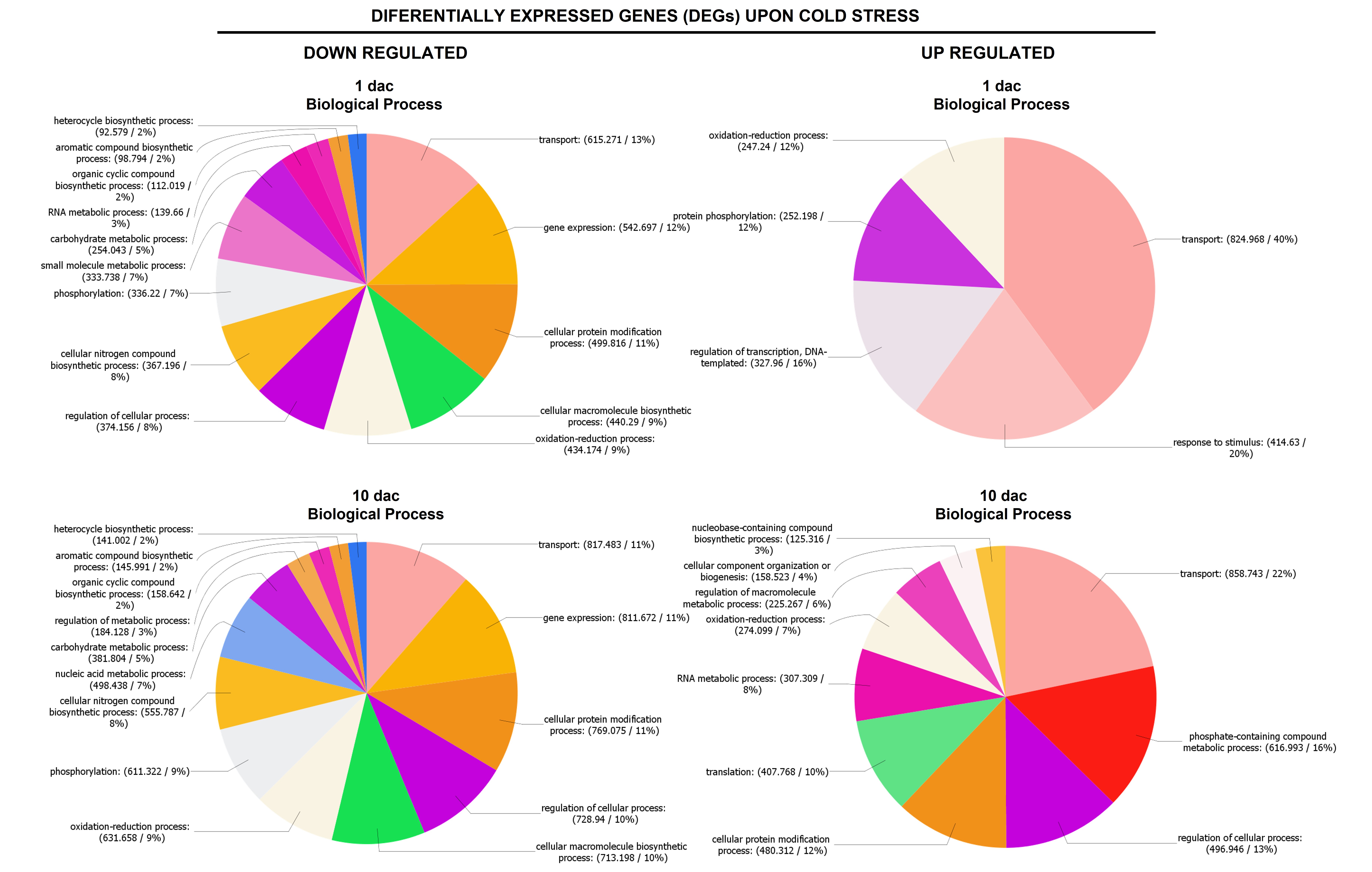

Supplement: Supplementary file 1 [file biology-11-01040-s001.zip › Supplementary FigS6.jpg]

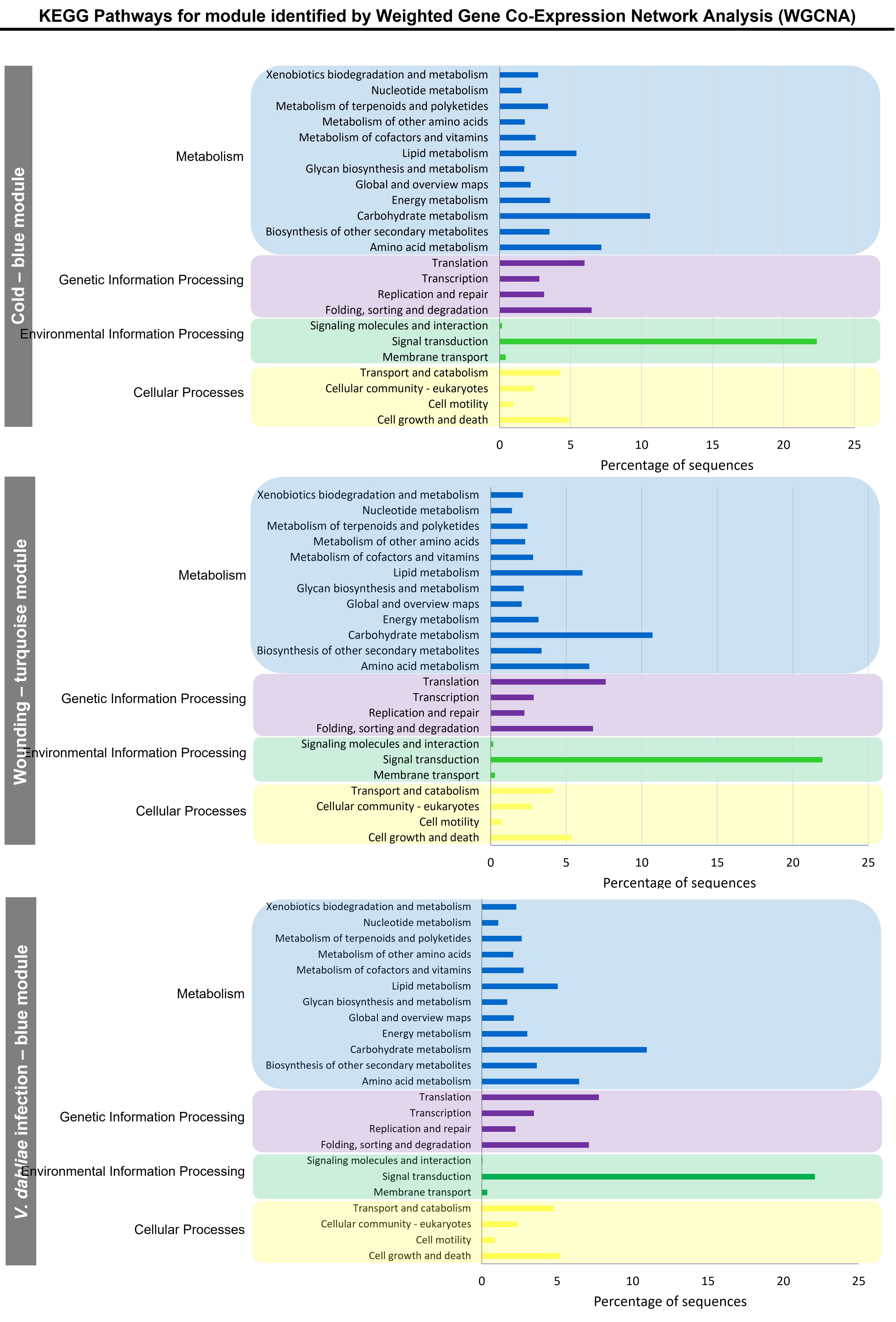

Supplement: Supplementary file 1 [file biology-11-01040-s001.zip › Supplementary FigS7.jpg]
